# Applications of hydrogels and nanoparticles in the treatment of traumatic brain injury

**DOI:** 10.3389/fbioe.2024.1515164

**Published:** 2025-01-06

**Authors:** Jiaying Shi, Jiajia Tang, Jin Xu, Ning Jiang, Yuanwei Yang, Honglin Chen, Yuhan Han, Xianhua Fu

**Affiliations:** ^1^ Department of Neurosurgery, The Affiliated Suqian First People’s Hospital of Nanjing Medical University, Suqian, China; ^2^ Department of Neurosurgery, Ren Ji Hospital, Shanghai Jiao Tong University School of Medicine, Shanghai, China; ^3^ Shanghai Jiao Tong University School of Medicine, Shanghai, China

**Keywords:** traumatic brain injury, hydrogel, nanoparticle, drug delivery, neuroprotection

## Abstract

Traumatic brain injury (TBI) represents a significant global public health issue, with effective management posing numerous challenges. The pathophysiology of TBI is typically categorized into two phases: primary and secondary injuries. Secondary injury involves pathophysiological mechanisms such as blood-brain barrier (BBB) disruption, mitochondrial dysfunction, oxidative stress, and inflammatory responses. Current pharmacological strategies often encounter obstacles in treating TBI effectively, primarily due to challenges in BBB penetration, inadequate target site accumulation, and off-target toxicity. Versatile hydrogels and nanoparticles offer potential solutions to these limitations. This review discusses recent progress in utilizing hydrogels and nanoparticles for TBI treatment over the past 5 years, highlighting their relevance to the underlying injury pathophysiology. Hydrogels and nanoparticles demonstrate substantial promise in addressing secondary brain injury, providing a broad spectrum of future therapeutic opportunities.

## 1 Introduction

Traumatic brain injury (TBI) is highly prevalent worldwide, resulting in a substantial public health burden. TBI is commonly caused by vehicular collisions or falls (Maas et al., 2022). While the etiology of TBI is diverse, the pathophysiological process is generally divided into two phases: primary injury and secondary injury. Primary injury typically results from the immediate impact of mechanical forces, whereas secondary injury develops from hours to years post-injury, encompassing blood-brain barrier (BBB) disruption, mitochondrial dysfunction, oxidative stress, and inflammatory responses (Thapa et al., 2021). Numerous studies have developed strategies to mitigate secondary injury, with some emerging materials being especially notable.

Multiple interventions exist for TBI, including pharmacotherapy, cognitive rehabilitation, and surgical procedures (Aqel et al., 2023). Nevertheless, there are currently just a few pharmacological therapies available for TBI due to the following challenges (Tani et al., 2022). First, medications may be obstructed by the BBB when targeting the brain, and nearly all macromolecular pharmaceuticals are unable to traverse the BBB (Yu et al., 2019; Wu et al., 2023a). Additionally, the distinctive pathophysiological characteristics of the brain present hurdles to the diffusion, distribution, and retention of medicines, hindering their accumulation to attain therapeutic concentrations (Nance et al., 2022; Wolak and Thorne, 2013). Furthermore, conventional pharmaceuticals are susceptible to accumulation in several organs, resulting in off-target adverse effects (Nance et al., 2022). The aforementioned problems restrict the utilization of conventional medication delivery techniques in TBI, hence highlighting the necessity to investigate novel materials for the treatment of secondary brain injury.

Hydrogels and nanoparticles are prominent emerging materials. Hydrogels are a category of water-absorbent three-dimensional polymer networks, typically classified into natural hydrogels and synthetic hydrogels (Cao et al., 2021; Xie and Xie, 2024). Nanoparticles are characterized as particles with dimensions between 1 and 1,000 nm (Bharadwaj et al., 2018). Hydrogels and nanoparticles provide substantial benefits in the management of brain diseases. First, they successfully traversed the BBB and accessed the cerebral lesions. The hydrogel can be administered by intranasal and intravenous methods, bypassing the BBB and directly accessing the brain (Xie and Xie, 2024). Based on their size or surface alterations, nanoparticles can improve targeted drug delivery by breaking tight junctions between endothelial cells or crossing the BBB through endocytosis (Zhou et al., 2018). Moreover, hydrogels and nanoparticles exhibit significant customization potential, allowing for adjustments to their properties—such as the mechanical and rheological characteristics of hydrogels, and the stability and particle size of nanoparticles—to enhance drug distribution and retention within the brain microenvironment (Saraiva et al., 2016; Öztürk et al., 2024). Furthermore, the stimulus-responsive characteristics of hydrogels and nanoparticles allow them to react to changes in the local microenvironment of brain lesions for precise medication administration and release (Xie and Xie, 2024).

Owing to the aforementioned benefits, hydrogels and nanoparticles have been employed in clinical trials for several neurological disorders. Magnetic resonance imaging with iron oxide nanoparticles has been shown to identify macrophage infiltration in the brain for disease detection and diagnosis (Dousset et al., 2006; Khan et al., 2019). A phase II clinical trial has shown that gadolinium-based nanoparticles can concentrate in brain malignancies and contribute to targeted and localized radiation (Bennett et al., 2024). Numerous nano-delivery technologies for Alzheimer’s disease treatment were being examined at different phases of clinical studies (Bahadur et al., 2020). Hydrogels and nanoparticles exhibit significant potential in the treatment of neurological disorders. These innovative materials also exhibit potential in TBI (Table 1). A phase IV trial established the effectiveness of transdermal testosterone gel in the recuperation from hypogonadism following TBI (Ripley et al., 2020). Other hydrogels are mostly used for dural repair in TBI surgery and are in different clinical stages (Green et al., 2015; Strong et al., 2017; Osbun et al., 2012). Nonetheless, the utilization of nanoparticles in TBI remains in the preliminary research phase and has yet to be extensively implemented in clinical settings. The utilization of hydrogel is similarly restricted in the clinical stage. Consequently, it is essential to examine the latest research on the utilization of these advanced materials in TBI, which will facilitate the advancement of fundamental research and future clinical applications.

**TABLE 1:** Clinical evidence for the application of hydrogel in traumatic brain injury.

Hydrogel	Application	Clinical phase	
Androgel	The rehabilitation of hypogonadism after TBI	Ⅳ (NCT01201863)	Ripley et al. (2020)
EVICEL^®^	Dural repair during skull surgery	Ⅲ (NCT02457546)	Green et al. (2015)
Adherus^®^	Dural repair during skull surgery	Approved by FDA	Strong et al. (2017)
DuraSeal^®^	Dural repair during skull surgery	Approved by FDA	Osbun et al. (2012)

TBI, traumatic brain injury; FDA, food and drug administration.

This review examines recent advancements over the past 5 years of *in vivo* research on the application of hydrogels and nanoparticles post-TBI, discussing their composition and efficacy. Their relationship with the pathophysiology of TBI is also highlighted.

## 2 Method

A comprehensive search of the prior research was conducted using the Web of Science database. For previous research related to hydrogel, our search term was as follows: {[(TS = “Traumatic brain injury”) AND (TS = “Hydrogel”)] AND (PY = 2020–2024)}. Up to 31 August 2024, a total of 142 publications were retrieved, and after selecting research articles and reviews, only 107 publications. For previous research related to nanoparticles, our search term was as follows: {[(TS = “Traumatic brain injury”) AND (TS = “Nanoparticle”)] AND (PY = 2020–2024)}. Up to 31 August 2024, a total of 168 publications were retrieved, and after selecting research articles and reviews, only 120 publications. Particularly, the most local cited publications, most global cited publications, and most global cited references were read with emphasis. This review was based on the work described above.

## 3 Mechanisms of TBI and challenges in pharmacological treatment

### 3.1 Mechanism and potential targets of TBI

Primary and secondary injury represent two phases in the pathophysiological progression of TBI. Primary injury includes mechanical brain injury, vascular damage, and hematoma development (Ng and Lee, 2019). It refers to the damage caused by the direct impact of external forces on the brain, typically resulting in immediate effects on the patient, which are often refractory or even fatal (Ng and Lee, 2019; Orr et al., 2024). Secondary brain injury occurs following the primary injury and involves acute inflammatory responses, vasospasm, brain tissue swelling, and worsening edema (Jullienne et al., 2016). During this phase, the BBB is disrupted, cerebrospinal fluid (CSF) circulation is impaired, and the intracranial microenvironment is altered, leading to persistent neurological dysfunction in TBI patients (Cash and Theus, 2020). Currently, clinical approaches to treating TBI remain relatively limited (Lucke-Wold et al., 2018). Besides conservative management, the focus for critically injured patients remains largely on early surgical intervention, with a lack of targeted treatments for secondary brain injury. Therefore, pharmacological treatment aimed at secondary brain injury is currently a focal point and challenge in the clinical management of TBI.

External forces exerted directly on brain tissue can result in mechanical damage or neuronal displacement, specifically axonal damage and synaptic impairment. These physical injuries primarily occur during the initial injury phase and can significantly impact brain function (Jamjoom et al., 2021). Secondary injury mechanisms following TBI, including oxidative stress and inflammatory responses, exacerbate neuronal damage. Free radicals and excitatory neurotransmitters like glutamate can induce lipid peroxidation and DNA damage in cell membranes, thereby accelerating neuronal death (Ng and Lee, 2019; Rauchman et al., 2023). Neuronal degeneration continues beyond the acute phase, with certain impaired neurons potentially undergoing apoptosis or necrosis, thus impacting the restoration of cerebral function (Akamatsu and Hanafy, 2020). Concurrently, healing mechanisms, including nerve regeneration and synaptic remodeling, are beneficial; however, they are often inadequate, resulting in prolonged neurological impairment (Coyne et al., 2006).

Oxidative stress is a major contributor to secondary injury following TBI (Ng and Lee, 2019). The brain is highly demanding in terms of oxygen and energy (Watts et al., 2018). After TBI, the self-repair processes of neurons require substantial amounts of oxygen and energy (Thapa et al., 2021), which disrupts the balance between energy supply and demand. As a result, large quantities of reactive oxygen species (ROS) are generated and accumulate within the post-injury microenvironment. Following TBI, endogenous ROS and free radicals persistently accumulate from multiple sources (Ng and Lee, 2019). Calcium influx results in mitochondrial dysfunction, ROS production, and suppression of free radical scavengers (Ng and Lee, 2019; Singh et al., 2006). The hypoxic condition of the brain and impaired mitochondria following vascular injury force cells to depend on glycolysis, leading to lactic acid buildup and resulting in aberrant energy metabolism (Patet et al., 2016; Zhou and Kalanuria, 2018). Simultaneously, increased ROS causes lipid peroxidation, leading to additional damage to the mitochondrial and cellular membranes (Thapa et al., 2021).

In the complex cascade of secondary injury, neuroinflammation is crucial in determining the prognosis of TBI (Visser et al., 2022). Previously, neuroinflammation following TBI was thought to result solely from peripheral immune mediators entering the central nervous system (CNS) through a compromised BBB. However, the prevailing view now recognizes that neuroinflammation after TBI involves a complex interaction between central and peripheral cells, as well as soluble factors (Simon et al., 2017). TBI can trigger early activation of resident microglia in the CNS, accompanied by the recruitment of peripheral neutrophils, followed by the infiltration of lymphocytes and monocyte-derived macrophages (Corps et al., 2015). Simultaneously, the sequential expression and secretion of pro-inflammatory and anti-inflammatory mediators can either promote or mitigate the neuroinflammatory response after TBI (Rodney et al., 2018). Chemokine-related signaling pathways play a key role, as they activate and recruit immune cells to the site of injury (Shi et al., 2019). Neuroinflammation after TBI is a double-edged sword: it has beneficial aspects, such as facilitating debris clearance and regeneration, but also mediates neuronal death and progressive neurodegeneration. However, excessive cytokine and chemokine secretion can disrupt the BBB, prolong the inflammatory process, and further exacerbate chronic neurodegeneration (Javalgekar et al., 2024). The various pathophysiological mechanisms of brain injury are interconnected, creating a vicious cycle that continuously exacerbates the damage (Ng and Lee, 2019). Therefore, developing targeted pharmacological interventions for different stages of TBI is of considerable clinical importance.

### 3.2 Challenges in pharmacological treatment of TBI

**FIGURE 1:**
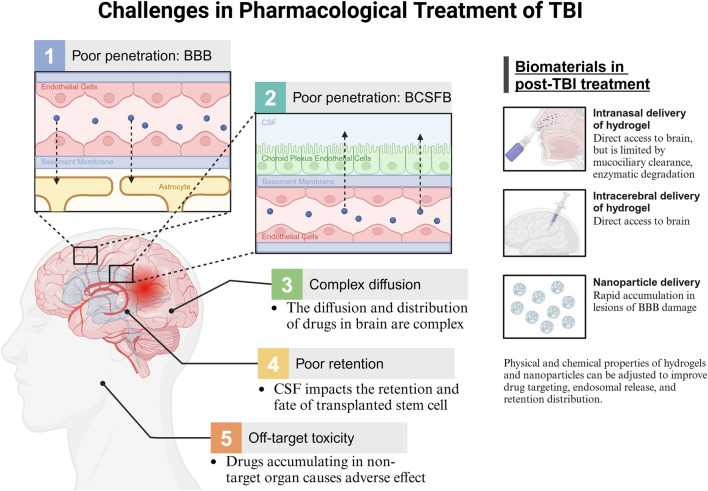
Challenges in pharmacological treatment of TBI. BBB, blood brain barrier; BCSFB, blood cerebrospinal fluid barrier; CSF, cerebrospinal fluid.

The prevailing approach to treating TBI involves oxygenation interventions, fluid management, hypothermia, and surgical procedures (Davis et al., 2024; Lewis et al., 2017). Many therapeutic approaches are limited by the complex pathophysiology associated with brain injury (Banderwal et al., 2024). Pharmacotherapy following TBI remains under investigation, and small molecule, peptide, or cell-based therapies are widely studied, although it faces numerous challenges. Figure 1 illustrates the challenges associated with pharmacological treatment following TBI, and the current biomaterial-based delivery routes.

Many medications have limited therapeutic efficacy in the brain due to insufficient drug delivery. The primary mechanism for controlling drug access to the brain is the selective permeability of the BBB. Brain microvascular endothelial cells are a crucial element of the BBB that block the passage of hydrophilic substances, charged molecules, proteins, and peptides, thereby limiting most medications from entering the brain (Xiong et al., 2021; Pulgar, 2018). Although TBI affects BBB integrity, BBB recovery after TBI does not resolve the challenges of drug delivery (Lee et al., 2019a). Moreover, drug transport to the brain is influenced by the blood-CSF barrier (BCSFB) (Nance et al., 2022). In contrast to the BBB, the BCSFB exhibits leakage, allowing certain molecules to traverse the choroid plexus and then passively diffuse from the CSF into brain tissue (Pardridge, 2011). However, further penetration of molecules into the brain parenchyma is restricted by limited CSF flow in the brain parenchyma (Nance et al., 2022).

Drug penetration into brain parenchyma remains a major challenge. The diffusion and distribution of drugs within the brain’s extracellular space are influenced by the brain microenvironment as well as the physical and chemical properties of the drug, such as size, surface charge, and shape (Nance et al., 2022; Wolak and Thorne, 2013). Heterogeneous extracellular space in various brain regions results in anisotropic diffusion, thereby complicating drug distribution (Nance et al., 2022).

Additionally, drugs have insufficient retention within the brain. Fluid shear stress produced by CSF circulation in the brain can affect the transplantation, viability, and differentiation of transplanted stem cells (Jing et al., 2021). Furthermore, the significant accumulation of systemically administered drugs in non-target organs leads to off-target toxicities and side effects (Lamade et al., 2019). Therefore, targeted approaches for drug administration are needed to minimize systemic side effects.

Current biomaterial applications offer potential solutions to these challenges. Hydrogels can be administered via intracranial or intranasal routes, bypassing the BBB and BCSFB for direct brain interaction (Marcello and Chiono, 2023). Intranasal delivery is limited by mucociliary clearance and enzymatic degradation (Formica et al., 2022). Nanoparticles promote rapid accumulation at the site of BBB damage (Saraiva et al., 2016). Additionally, the tunable physical and chemical properties of hydrogels and nanoparticles can be adjusted to improve drug targeting, endosomal release, and retention within brain tissue.

## 4 Application of hydrogels post-TBI

### 4.1 Properties and types of hydrogels and their implications for applications in TBI

#### 4.1.1 Mechanical and rheological properties of hydrogels used in TBI treatment

In the management of TBI, hydrogels must possess sufficient strength to sustain the local tissue architecture, while ideally conforming to the rheological properties of brain tissue to minimize stimulation of the injured cerebral tissue. Consequently, suitable mechanical and rheological qualities significantly influence the effectiveness of hydrogels in TBI.

The elastic modulus is a crucial mechanical parameter of hydrogels, indicating their capacity to withstand elastic deformation under stress (Lee et al., 2019b; Subramanian, 2020). The tensile elastic modulus (Young’s modulus) of cerebral tissue is around 1 kPa (Gefen and Margulies, 2004). Creating hydrogels that match the stiffness of brain tissue is essential for determining cell fate (Cao et al., 2021).

Rheological qualities refer to the flow and deformation characteristics of hydrogels subjected to varying shear forces. The storage modulus (G′) indicates the elastic properties of hydrogels, whereas the loss modulus (G″) denotes their viscous properties (Baby et al., 2020). The G′ of human brain tissue varies between 140 and 620 Pa (Rowland et al., 2018). If the hydrogel modulus is comparable, the hydrogel can maintain a secure adhesion to the brain tissue.

#### 4.1.2 Types and scaffolds of hydrogels

Hydrogels can be categorized into natural and synthetic types based on their polymeric foundations.

Bio-based scaffolds such as collagen, gelatin, hyaluronic acid (HA), alginate, and chitosan are commonly employed as the foundational components of natural hydrogels (Cao et al., 2021). Biopolymers generally demonstrate exceptional biocompatibility, effectively replicating the tissue’s milieu and provoking suitable biological reactions. For instance, HA in the brain can impede neural scar formation and activate endothelial cell receptors to promote angiogenesis (Lainé et al., 2022). Meanwhile, most biopolymers are biodegradable and can be decomposed by enzymes like collagenase and matrix metalloproteinases (MMPs) (Cao et al., 2021). Furthermore, chitosan, alginate, and HA exhibit antibacterial and anti-inflammatory properties (Catoira et al., 2019). Nonetheless, inferior mechanical qualities represent a considerable limitation in the utilization of biopolymer hydrogels. Researchers frequently integrate nanoparticles or microparticles into hydrogels or utilize cross-linking to improve the mechanical properties of biopolymer hydrogels (Zhang et al., 2018a).

Synthetic polymers, such as polyethylene glycol and polypropylene, are commonly utilized as scaffolds in synthetic hydrogels. These scaffolds possess significant mechanical strength; yet, they lack biocompatibility and biological activity (Cao et al., 2021). Researchers typically attach MMP to synthetic polymers via modification, rendering synthetic hydrogels biodegradable (Della Sala et al., 2020).

Different scaffolds are linked to distinct property traits. Table 2 outlines the physical characteristics and affecting elements of various hydrogel substrates. The mechanical and rheological properties of the final hydrogel products can be tailored to brain tissue by the relevant contributing elements.

**TABLE 2:** Properties of different scaffolds.

	Composition	Mechanical property	Rheological property	
Bio-based				
Alginate	β-D-mannuronate (M) and α-L-hepturonate (G)	- Weak - Related to number and distribution of G blocks (which form ion Bridges), crosslink	- Related to MW, the cation concentration of cross-linking agent (Ca^2+^, etc.)	Petersen et al. (2023) and Cuomo et al. (2019)
Chitosan	D-glucosamine and N-acetyl-D-glycosamine groups	- Weak - Related to MW, degree of deacetylation, crosslink	- Related to MW, pH, electrolyte, the degree of deacetylation, the addition of alcohol solvent	Subramanian (2020), Richa and Choudhury (2020), and Budai et al. (2023)
Collagen	The primary structure is Gly-X-Y	- Weak - Related to temperature, pH, and crosslink	- Related to temperature, MW, pH	Lian et al. (2016) and Sarrigiannidis et al. (2021)
Gelatin	Triple helix structure of (Gly-X-Pro) n	- Weak - Related to MW, the number of helical structure, crosslink	- Related to MW, pH, electrolyte, and enzyme treatment	Ahmed et al. (2023)
Hyaluronic acid	Glucuronic acid and N-acetylglucosamine	- Weak - Related to crosslink, modification	- Related to MW, the crosslink concentration, derivatization types	Chernos et al. (2017), Rebenda et al. (2020), Zerbinati et al. (2021), and Luo et al. (2023)
Synthetic				
Polypropylene	—	- Strong - Related to MW, modification, prepolymer concentration	- Related to MW, modification	Killion et al. (2012)

MW, molecular weight.

### 4.2 Promote neural regeneration and facilitate stem cell therapy

Stem cell therapy can stimulate neuronal regeneration and has paracrine effects, making it a viable treatment option for TBI (Dekmak et al., 2018). However, the survival rate of transplanted cells is low, and neural differentiation is limited at the site of TBI (Coyne et al., 2006). Hydrogels are a viable means of delivering stem cells, which can improve the survival and targeted implantation of stem cells (Kim et al., 2023). Here we reviewed previous studies that utilized hydrogel technology with stem cell therapy to enhance engraftment and efficacy.

**FIGURE 2:**
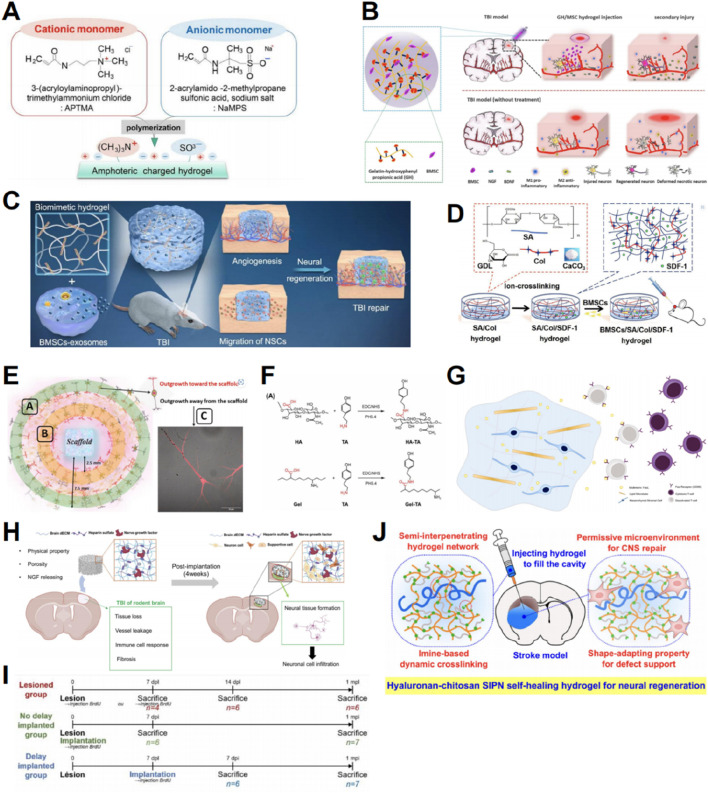
Graphic abstracts of the current hydrogel applications in promoting neural regeneration and facilitating stem cell therapy. **(A)** Amphoteric charged hydrogel, (reprinted with permission from Tanikawa et al., 2023, ©2023 by the authors); **(B)** A gelatin hydrogel to load BMSCs, (reprinted with permission from Li et al., 2021, ©2021 Elsevier B.V.); **(C)** A hyaluronan hydrogel to load BMEs, (reprinted with permission from Liu et al. , 2023a, ©2023 Elsevier Ltd); **(D)** An alginate/collagen/SDF-1 hydrogel to load BMSCs, (reprinted with permission from Ma et al., 2021, ©2021 Acta Materialia Inc); **(E)** Evaluating the nerve regeneration ability of the prepared hydrogel, (reprinted with permission from Mishchenko et al., 2022, ©2022 by the authors); **(F)** Partial steps in the synthesis of HA/Gel, (reprinted with permission from Zhou et al., 2024, ©2024 by the authors); **(G)** Immune-regulated hydrogels to load MSCs, (reprinted with permission from Alvarado-Velez et al., 2021, ©2020 Elsevier Ltd); **(H)** ECM-based cryogels to load nerve growth factors and heparin sulfate, (reprinted with permission from Kim et al., 2024, ©2024 Elsevier B.V.); **(I)** Timeline of the mice hydrogel implantation and sacrifice, (reprinted with permission from Lainé et al., 2022, ©2022 by the authors); **(J)** Hyaluronan-chitosan hydrogels, (reprinted with permission from Liu et al., 2020, ©2020, American Chemical Society).

Neural stem cells (NSCs) are commonly used in TBI treatment (Kim et al., 2023). Kim et al. (2023) proposed a novel delivery method for mouse NSC spheroids using hydrogels, demonstrating effective engraftment and cell survival. Chen et al. (2023) identified CSF flow after TBI as an important factor affecting cell loss after NSC transplantation and integrated gelatin methacrylate/sodium alginate hydrogel scaffolds with pre-differentiated NSCs to mitigate CSF flow-related cell loss. Tanikawa et al. (2023) found that an equal ratio of anionic and cationic porous hydrogel can serve as a scaffold for effective NSC attachment, promoting the differentiation of NSC into glial and neuronal cells (Figure 2A).

Aside from NSCs, mesenchymal stem cells (MSCs), derived from various sources, can differentiate into neural cells in lesions to substitute impaired or missing neurons, making them a promising candidate for stem cell therapy (Kariminekoo et al., 2016). Wang et al. (2022a) incorporated bone marrow mesenchymal stem cells (BMSCs) and nerve growth factors into tyramine-modified HA (HT) hydrogels to promote the regeneration of impaired brain tissue. Li et al. (2021) created a gelatin-hydroxyphenyl hydrogel cross-linked with horseradish peroxidase and choline oxidase to load BMSCs. The results showed that the hydrogel could significantly promote cell viability, neural differentiation, and secretion of neurotrophic factors by loaded BMSCs, thereby enhancing the therapeutic effect of BMSCs in mice (Figure 2B) (Li et al., 2021). An innovative approach for integrating BMSC-derived exosomes (BME) into HA-collagen hydrogel was proposed by Liu et al. (2023a). This approach demonstrated the induction of angiogenesis and neurogenesis, recruitment of endogenous NSCs for neuronal differentiation, and facilitation of vascularization (Figure 2C) (Liu et al., 2023a).

It is worth noting that in the above experiments we mentioned, the hydrogel developed by Tanikawa et al. (2023) and the hydrogel invented by Liu et al. (2023a), differ substantially in terms of Young’s modulus, ranging from 1.6 to 133.8 kPa for the former and from 0.6 to 0.8 kPa for the latter. More interestingly, the former enhanced NSC differentiation into neurons and glial cells (Tanikawa et al., 2023), while the latter promoted NSC differentiation into neurons (Liu et al. 2023a). This totally reveals that Young’s modulus has a considerable influence on the cell fate of NSC. Indeed, the aforementioned discrepancies are in keeping with earlier findings, where NSC is prone to neuronal differentiation in stiffness ≈0.1–1 kPa and glial differentiation in stiffer materials (Tseng et al., 2015). This further highlights the power of hydrogels to impact the result of TBI cell therapy through the change of their mechanical characteristics.

Hydrogels can also be used to transport certain factors that affect repair after TBI, thereby enhancing their biological activity and enabling controlled release. Stromal cell-derived factor-1 (SDF-1) and its receptor CXCR4 play crucial roles in regulating stem cell survival, recruitment, and differentiation (Shichinohe et al., 2007). An alginate/collagen/SDF-1 gel loaded with BMSCs was investigated by Ma et al. (2021) This gel was shown to enhance the survival, migration, and neuronal differentiation of BMSCs in lesions by activating the SDF-1/CXCR4-mediated FAK/PI3K/AKT pathway (Figure 2D) (Ma et al., 2021). By integrating recombinant SDF-1 protein into self-assembled peptide hydrogels, Wang et al. (2022b) created an environment supportive of transplanted cell survival. Other factors can also be delivered to the TBI site via hydrogels to promote regeneration and repair. Mishchenko et al. (2022) produced HA scaffolds impregnated with neurotrophic factors that demonstrated regenerative potential in in *vivo* experiments (Figure 2E). Developed by Ma et al. (2020), the self-assembling peptide-based hydrogel incorporating a mimic of vascular endothelial growth factor (VEGF-165) demonstrated significant repair capabilities. Zhou et al. (2024) formulated an injectable hydrogel consisting of HA and gelatin, combined with salvianolic acid B and vascular endothelial growth factor (Figure 2F).

Several challenges persist in stem cell therapies. One challenge is the premature death of exogenous stem cells due to immune rejection at the injury site. By transporting proteins that regulate the immune response at the transplantation site, hydrogels can enhance stem cell treatment. The protein FasL is a pro-apoptotic mediator that interacts with the Fas receptor found on the outer membrane of different immune cells to eliminate excess immune cells (Siegel et al., 2000). Alvarado-Velez et al. (2021) investigated the simultaneous administration of FasL and MSC using a hydrogel to establish an immunosuppressive milieu and decrease the local cytotoxic CD8 T cell population, thus enhancing the survival of transplanted MSCs (Figure 2G).

Another challenge for stem cell therapy is the formation of nerve scars after brain injury, which impedes the growth of normal neuronal cells (Kim et al., 2024; Liu and Hsu, 2020). Hydrogels provide mechanical benefits by occupying the lesion cavity and preventing nerve scar formation, thereby facilitating cell proliferation and differentiation (Liu and Hsu, 2020). Hydrogels associated with the extracellular matrix (ECM) can replicate the natural brain environment. Kim et al. (2024) developed decellularized ECM incorporated with nerve growth factors and heparin sulfate-based cryogels, which significantly promoted brain tissue regeneration (Figure 2H). Natural ECM-derived HA inhibits glial scar formation (Khaing and Seidlits, 2015; Lin et al., 2009). Lainé et al. (2022) demonstrated that implanted HA hydrogels provide a supportive environment for the survival and maturation of newly generated neurons (Figure 2I). Liu et al. (2020) integrated HA into chitosan-based self-healing hydrogels to create an adaptive environment that supports the spreading, migration, proliferation, and differentiation of NSCs (Figure 2J).

### 4.3 Hydrogel can facilitate ROS scavenging post-TBI

**FIGURE 3:**
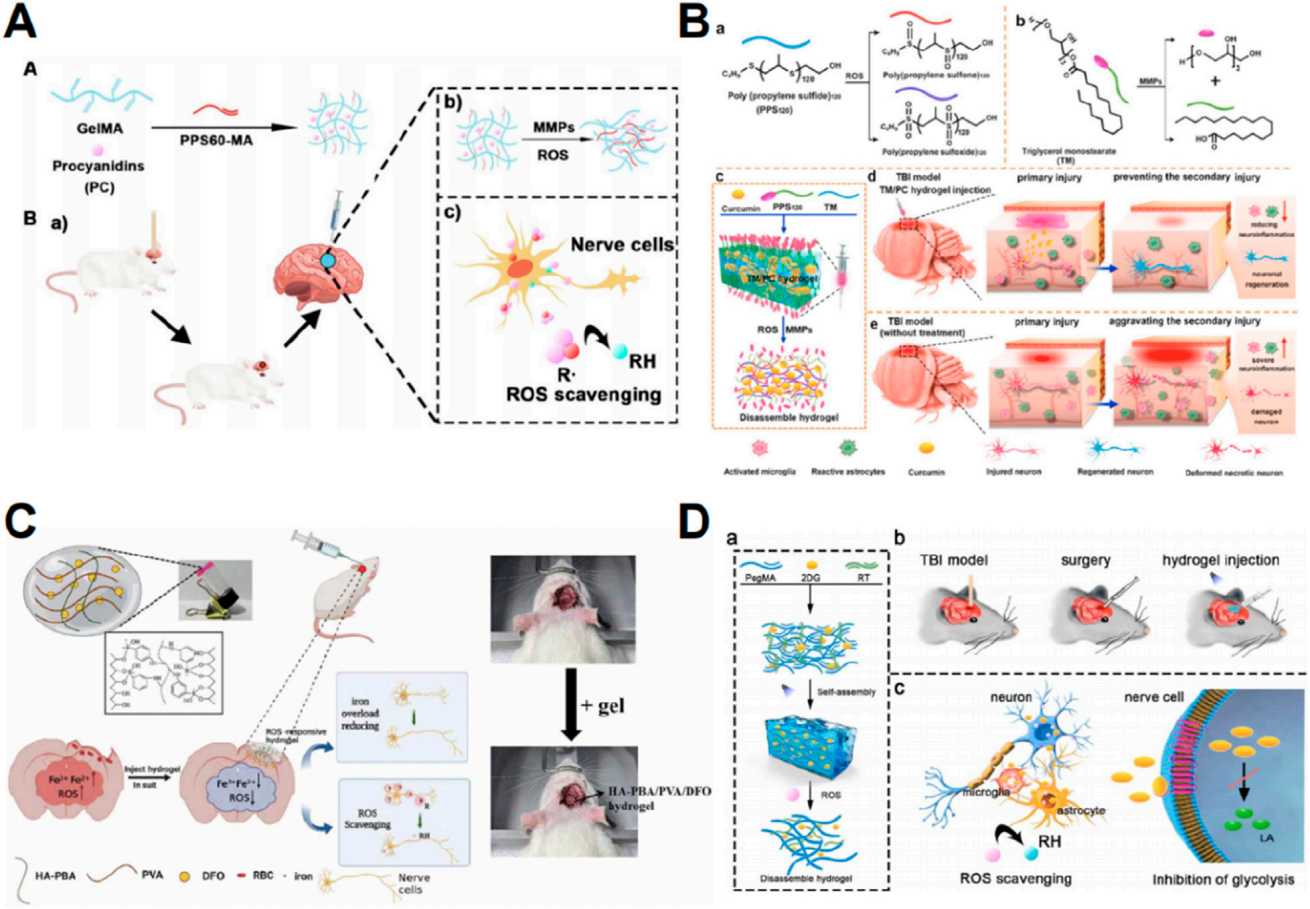
Graphic abstracts of the current hydrogel applications in scavenging reactive oxygen species post traumatic brain injury. **(A)** The GelMA-PPS/PC hydrogel, (reprinted with permission from Huang et al., 2022, ©2022, American Chemical Society); **(B)** The TM/PC hydrogel, (reprinted with permission from Qian et al., 2021, ©2021 Elsevier Ltd); **(C)** The HA-PBA/PVA/DFO hydrogel, (reprinted with permission from Qiu et al., 2024, ©2024 IOP Publishing Ltd); **(D)** The P-RT/2DG hydrogel, (reprinted with permission from Han et al., 2024a, ©2024 The Authors).

After TBI, ROS accumulates in the traumatic microenvironment, leading to numerous secondary brain injuries, compromising the integrity of the BBB, and aggravating brain edema (Fesharaki-Zadeh, 2022). Many studies have explored the role of hydrogels in eliminating ROS, and their therapeutic efficacy for TBI has been demonstrated *in vivo*. Hydrogels have been utilized as carriers for ROS quenchers. In the presence of ROS, hydrophobic poly (propylene sulphide) (PPS) can undergo oxidation to become hydrophilic, thus facilitating the controlled release of drugs in an oxidative environment (Gupta et al., 2014). Huang et al. (2022) employed gelatin methacrylate and PPS60 loaded with proanthocyanidins, potent antioxidants, to efficiently reduce ROS levels, decrease brain edema, preserve BBB integrity, alleviate neuroinflammation, and facilitate functional recovery in subjects with secondary injury (Figure 3A). Qian et al. (2021) formulated hydrogels containing triglycerol monostearate, PPS120, and curcumin (an antioxidant) and verified their ability to reduce ROS, mitigate neuroinflammation, and promote neuronal regeneration and functional recovery following secondary injury (Figure 3B).

Prolonged iron buildup in the brain following TBI causes lipid peroxidation and the production of ROS (Tang et al., 2020). Therefore, a potential strategy is to prevent excessive iron accumulation and consequent oxidative stress. When administered orally or intravenously, deferoxamine mesylate (DFO) is a well-known iron chelator (Holden and Nair, 2019). However, it has limited ability to cross the BBB and exhibits non-specific toxicity at high dosages (Qiu et al., 2024). Hydrogels represent an efficient platform for the targeted delivery of DFO. To reduce iron overload at TBI lesions and eliminate ROS, Qiu et al. (2024) developed a hydrogel by grafting HA and polyvinyl alcohol with phenylboric acid to release DFO (Figure 3C).

Following TBI, a significant buildup of ROS causes mitochondrial dysfunction, resulting in an elevation of glycolytic lactate levels (Zhou and Kalanuria, 2018; Khatri et al., 2018). These metabolic abnormalities exacerbate oxidative stress (Han et al., 2024a). Thus, interrupting the harmful cycle of oxidative stress and glycolysis could effectively reduce ROS levels and mitigate subsequent damage caused by TBI. A glucose analog, 2-deoxyglucose (2DG), can suppress glycolysis (Pajak et al., 2019), but the practical use of 2DG in clinical settings is restricted due to its extensive harmful impact on non-target cells and its requirement for high concentrations (Figure 3D) (Han et al., 2024a). These challenges can be overcome by the implementation of targeted administration and ROS-responsive hydrogel systems. Han et al. (2024a) employed poly (ethylene glycol) dimethacrylate and a ROS-responsive thiothiol linker to develop hydrogels that demonstrate targeted 2DG release, as well as inhibition of ROS and lactate production.

Much of the aforementioned research concentrated solely on the removal of ROS; however, the mechanism of oxidative stress following TBI includes not only ROS production but also the generation of reactive nitrogen species (RNS). Calcium ion buildup during TBI enhances nitric oxide synthesis via nitric oxide synthase, subsequently leading to oxidative damage (Kaur and Sharma, 2018). Future research may explore the utilization of RNS as a novel target to mitigate oxidative stress in cerebral tissue following TBI.

### 4.4 Hydrogel can promote anti-inflammation post-TBI

Following TBI, astrocytes and microglia in the lesions become stimulated, leading to the secretion of several inflammatory cytokines and chemokines (Mira et al., 2021). Although these inflammatory reactions help the regeneration of certain tissues, too intense inflammatory responses can result in neuronal cell injury, disruption of the BBB, swelling, and further cell death (Schimmel et al., 2017). Hence, it is crucial to regulate the inflammatory reaction in patients with TBI in order to minimize residual damage.

**FIGURE 4:**
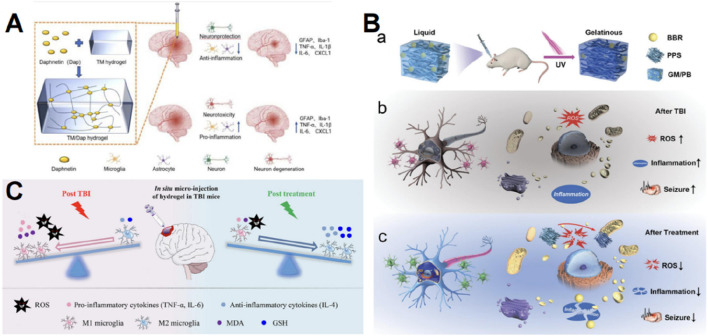
Graphic abstracts of the current hydrogel applications in anti-inflammation therapy post traumatic brain injury. **(A)** The TM/Dap hydrogel, (reprinted with permission from Ma et al., 2024, ©2024 The authors); **(B)** The GM/PB hydrogel, (reprinted with permission from Han et al., 2024b, ©2024 Wiley‐VCH GmbH); **(C)** The HT/HGA hydrogel, (reprinted with permission from Zhang et al., 2022, ©2022 The Authors).

Some anti-inflammatory medications have restricted effectiveness in managing inflammation following TBI because of their poor bioavailability or inability to penetrate the BBB (Javed et al., 2022; Jones et al., 2023). The development of hydrogels compensates for this constraint. Daphnetin possesses anti-inflammatory properties, enhances the integrity of the BBB, and decreases brain edema, but has poor bioavailability (Javed et al., 2022). In order to significantly enhance the therapeutic efficacy of daphnetin, Ma et al. (2024) employed tripolycerol monstearate as an encapsulant, which effectively reduced the neuroinflammatory effect (Figure 4A). Dexamethasone, a type of steroidal anti-inflammatory medication, alleviates neuroinflammation by targeting activated microglia and invading macrophages (Jones et al., 2023). Jones et al. (2023) and Macks et al. (2022) introduced a hydrolyzable hydrogel composed of poly (ethylene) diol-bis-(acryloxyacetate) and coupled with dexamethasone HA. Experimental evidence showed that the hydrogel containing dexamethasone effectively decreased neuroinflammation, apoptosis, and lesion size, while enhancing the survival of neuronal cells and the restoration of motor function (Jones et al., 2023; Macks et al., 2022). Decellularized ECM-derived biomaterials possess significant potential to mitigate inflammatory reactions (Wassenaar et al., 2016). Diaz et al. (2023) formulated a decellularized-ECM hydrogel to attenuate the proinflammatory response through intravenous administration into brain lesions.

Pyroptosis, a regulated cell death, that significantly activates intense neuroinflammation and amplifies the inflammatory response by releasing inflammatory contents, is greatly associated with inflammation activated by TBI (Simon et al., 2017). Hydrogen sulfide was shown to reduce cell death after TBI (Zhang et al., 2014). However, the development of nonvolatile, locally delivered, and low-dose exogenous H_2_S donors remains a challenge (Chen et al., 2022). A surface-filled H_2_S-releasing silk fibroin hydrogel was developed by Chen et al. (2022) with the aim of inhibiting neuronal pyroptosis, mitigating neurodegeneration and brain oedema, facilitating neurological functional recovery, and minimizing tissue loss and persistent neuroinflammation.

The previously described oxidative stress and the inflammatory response form a self-perpetuating loop, where the buildup of ROS generates a harmful milieu for brain tissue, leading to the release of numerous inflammatory signals (Abdul-Muneer et al., 2015). Hence, the aforementioned antioxidant hydrogel measures can also serve as anti-inflammatory agents, and additionally, the anti-inflammatory hydrogel can partially mitigate ROS. Berberine exhibits strong anti-inflammatory properties (Zhang et al., 2021). Han et al. (2024b) created an injectable gelatin methacrylate hydrogel to administer PPS60 and berberine for long-lasting and efficient therapy in juvenile TBI rats by decreasing ROS and neurotoxic inflammation (Figure 4B). Gallic acid acts by scavenging ROS and decreasing the release of inflammatory stimuli (Bai et al., 2021). Zhang D and colleagues grafted HA with gallic acid and integrated them into HT hydrogels, and ultimately the antioxidant hydrogel showed a distinct beneficial impact on suppressing oxidative stress and pro-inflammatory reactions, thus facilitating the restoration of motor, learning, and memory capabilities in mice with TBI (Figure 4C) (Zhang et al., 2022). The collaboration between antioxidants and anti-inflammatory drugs presents a promising opportunity to amplify therapeutic effects, and this dual mechanism is crucial for disrupting the detrimental loop of oxidative stress and inflammation.

### 4.5 Other applications of hydrogel post-TBI

In addition to the studies mentioned above, hydrogels have numerous additional applications in TBI treatment. Han et al. (2024c) combined hydrogel with hypothermia therapy, and the hydrogel they developed maintained hypothermia in the brains of TBI mice for 12 h without affecting systemic body temperature. The BBB remained intact at this reduced temperature, thereby reducing inflammation and brain edema (Han et al., 2024c).

Hydrogels can also provide strong adhesive and mechanical properties to fill the lesion cavity and stop bleeding. Dong et al. (2024) developed a robust adhesive and hemostatic hydrogel by crosslinking oxidized sodium alginate and carboxymethyl chitosan with calcium ions. Hu et al. (2023) developed a hydrogel utilizing phenylboronic acid-grafted HA and dopamine-grafted gelatin, demonstrating tissue adhesion, self-healing, and hemostatic capabilities upon injection into brain lesion cavities.

### 4.6 Special properties for injectable hydrogels

Table 3 encapsulates the aforementioned hydrogels, the majority of which exert their effects directly on the lesion via cerebral injection. For injectable hydrogels, characteristics such as self-healing and shear-thinning are crucial.

**TABLE 3:** Application of hydrogel post-TBI.

Main composition/biomaterial	Crosslink	In vivo models	Administration	
Neural protection and regeneration				
mNSC, collagen, fibrin	—	Male C57BL/6 mice	Intracerebrally	Kim et al. (2023)
NSC, gelatin-methacrylate hydrogel, sodium alginate	Physical	Male SD rats	Intracerebrally	Chen et al. (2023)
NSC, C1A1 hydrogel	Chemical	C57BL/6JJcl or NOD/Shi Jic-scid mice	Intracerebrally	Tanikawa et al. (2023)
BMSC, NGF, tyramine-modified HA	Chemical	Male C57BL/6 mice	Intracerebrally	^a^ Wang et al. (2022a)
BMSC, gelatin-hydroxyphenyl hydrogel	Chemical	Male C57BL/6 mice	Intracerebrally	Li et al. (2021)
BME, DHC	Chemical and physical	Male SD rats	Intracerebrally	Liu et al. (2023a)
BMSC, sodium alginate/collagen type I/SDF-1	Physical	Male SD rats	Intracerebrally	Ma et al. (2021)
SDF-1, self-assembly peptide	—	Swiss mice	Intracerebrally	Wang et al. (2022b)
HA, neurotrophic factors (BDNF, GDNF)	Chemical	Male C57BL/6 mice	Intracerebrally	Mishchenko et al. (2022)
Angiogenic self-assembling peptide-based hydrogel	Physical	Male SD rats	Intracerebrally	^a^ Ma et al. (2020)
HA, gelatin, VEGF, salvianolic acid B	Chemical	Male C57BL/6 mice	Intracerebrally	Zhou et al. (2024)
FasL-agarose hydrogels, MSC	Physical	Male SD rats	Intracerebrally	Alvarado-Velez et al. (2021)
Decellularized extracellular matrix, NGF, heparin sulfate-based cryogels	Chemical	Mice	Intracerebrally	^a^ Kim et al. (2024)
HA, other ECM components	Chemical	C57BL/6 mice	Intracerebrally	Lainé et al. (2022)
Chitosan-HA hydrogels	Chemical	Zebrafish, male SD rats	Intracerebrally	^b^ Liu et al. (2020)
ROS scavenging				
Gelatin methacrylate, poly (propylene sulfide)_60_, Procyanidins	Chemical	Male ICR mice	Intracerebrally	Huang et al. (2022)
TM, poly (propylene sulfide)_120_, Curcumin	—	Male ICR mice	Intracerebrally	Qian et al. (2021)
Deferrioxamine mesylate, phenylboronic acid grafted *HA*, polyvinyl alcohol	Chemical	Male SD rats	Intracerebrally	^a^ ^,^ ^b^ Qiu et al. (2024)
2-Deoxyglucose, poly (ethylene glycol) dimethacrylate, ROS-responsive thioketal linker	Chemical	Male ICR mice	Intracerebrally	Han et al. (2024a)
Anti-inflammation				
Daphnetin, tripolycerol monstearate	Physical	Male ICR mice	Intracerebrally	Ma et al. (2024)
Dexamethasone-conjugated HA, poly (ethylene) glycol-bis-(acryloyloxy acetate)	Chemical	Male SD rats	Intracerebrally	Jones et al. (2023) and Macks et al. (2022)
Cardiac-derived infusible extracellular matrix-derived biomaterial	—	C57BL/6 mice	Intravenously	^a^ Diaz et al. (2023)
H_2_S, silk fibroin hydrogel	Physical	Male ICR mice	Intracerebrally	Chen et al. (2022)
Berberine, poly (propylene sulfide)60, gelatin methacrylate hydrogel	Chemical	Rats	Intracerebrally	Han et al. (2024b)
HA grafted with gallic acid, HT	Chemical	Male C57BL/6 mice	Intracerebrally	Zhang et al. (2022)

NSC, nerve stem cell; BME, bone marrow mesenchymal stem cell (BMSC)-derived exosomes; NGF, nerve growth factor; HA, hyaluronic acid; DHC, hyaluronic acid modified by aldehyde groups and methacrylate (DHA) collagen; SD, Sprague-Dawley; SDF-1, Stromal cell-derived factor-1; VEGF, vascular endothelial growth factor.

^a^ hydrogel with shear-thinning property.

^b^ hydrogel with self-healing property.

Self-healing denotes the capacity of hydrogels to autonomously regain structural integrity and functionality following damage (Karvinen and Kellomäki, 2022; Wu et al., 2023b). The self-healing characteristics of hydrogels guarantee their efficacy post-injection and enhance their stability inside tissues, as hydrogels might experience mechanical injury during injection and subsequent interactions with adjacent tissues. Self-healing of the hydrogels developed by Qiu et al. (2024) is accomplished via dynamic boron-ester linkage. The hydrogels developed by Liu et al. (2020) are cross-linked by dynamic imine linkages and exhibit self-healing capabilities.

Besides self-healing, a significant characteristic of injectable hydrogels is shear-thinning, which refers to the reduction in viscosity of a hydrogel as the shear rate escalates (Chen et al., 2017). The shear-thinning feature enables the hydrogel to function as a less viscous substance for effortless injection, while maintaining its gel structure post-injection (Chen et al., 2017; Loebel et al., 2017). The shear thinning property is typically attributed to a reversible cross-linked structure inside the internal network of hydrogels, wherein viscosity diminishes as shear force disrupts weak links and recovers with the removal of shear force (Rizzo and Kehr, 2021). In the aforementioned hydrogels for TBI treatment, the engineered hydrogel developed by Ma X et al. can liquefy at a rate of 15 rad/s under high-frequency shear and can recover over 95% of the storage modulus within seconds following a shift from high to low stress. This pronounced shear-thinning characteristics are ascribed to its physical cross-linking via hydrophobic contacts and hydrogen bonds, facilitating facile dissociation and recombination (Ma et al., 2020; Kumar et al., 2015). The hydrogel created by Qiu et al. (2024) can regenerate from fractures reversibly in response to changes in stress by dynamically reversible boron-ester bond cross-linking. In addition, the hydrogels developed by Wang et al. (2022a), Kim et al. (2024), and Diaz et al. (2023), described above also exhibit shear-thinning properties.

## 5 Application of nanoparticle post-TBI

### 5.1 Properties of nanoparticles and the implications for applications in TBI

To ensure nanoparticles effectively contribute to the therapy of TBI, their stability, stimulatory reactivity, and particle size must be considered.

The zeta potential indicates the stability of the nanoparticles. The zeta potential signifies the surface charge of the nanoparticle; a larger absolute value denotes more electrostatic repulsion among the particles and reduced aggregation, hence indicating greater nanoparticle stability (Wang et al., 2023). Nanoparticles exhibiting zeta potentials exceeding +30 mV or falling below −30 mV in the aqueous phase were deemed generally stable (Honary and Zahir, 2013). It is important to note that significantly positive NP (+45 mV) resulted in rapid toxicity due to BBB breakdown (Lockman et al., 2004).

The dimension of nanoparticles also affected their efficacy on TBI. The nanoparticle size influences its pharmacokinetics and biodistribution in humans (Bharadwaj et al., 2018). Nanoparticles measuring between 20 and 100 nm are optimal for cerebral distribution, as they effectively traverse the BBB while exhibiting reduced renal clearance (Bharadwaj et al., 2016; Zhang et al., 2020).

The responsiveness of nanoparticles to stimuli is crucial for targeted medication release in reaction to local environmental alterations in brain tissue following TBI. Post-TBI, the locus of cerebral damage typically exhibits a diminished pH (Timofeev et al., 2013), so nanoparticles can release drugs in response to the local acidic environment. Takahashi et al. (2020) utilized pH-responsive nanoparticles to facilitate tailored drug release, as previously discussed. The amino groups within the nanoparticles can undergo protonation in an acidic pH environment, resulting in the collapse of the nanocage and the subsequent release of the internal medication (Takahashi et al., 2020). Moreover, photo-responsive nanoparticles can make alterations following irradiation with certain light wavelengths, thereby influencing medication release (Martín Giménez et al., 2021). The nanoparticles examined by Black et al. (2020) exhibited photo-responsiveness, and the caged nanoparticles will be disintegrated by a photo-redox process to liberate the active medication utilizing near-infrared light capable of penetrating bone and tissue (Black et al., 2020).

### 5.2 Nanoparticles can promote neural regeneration and facilitate stem cell therapy

**FIGURE 5:**
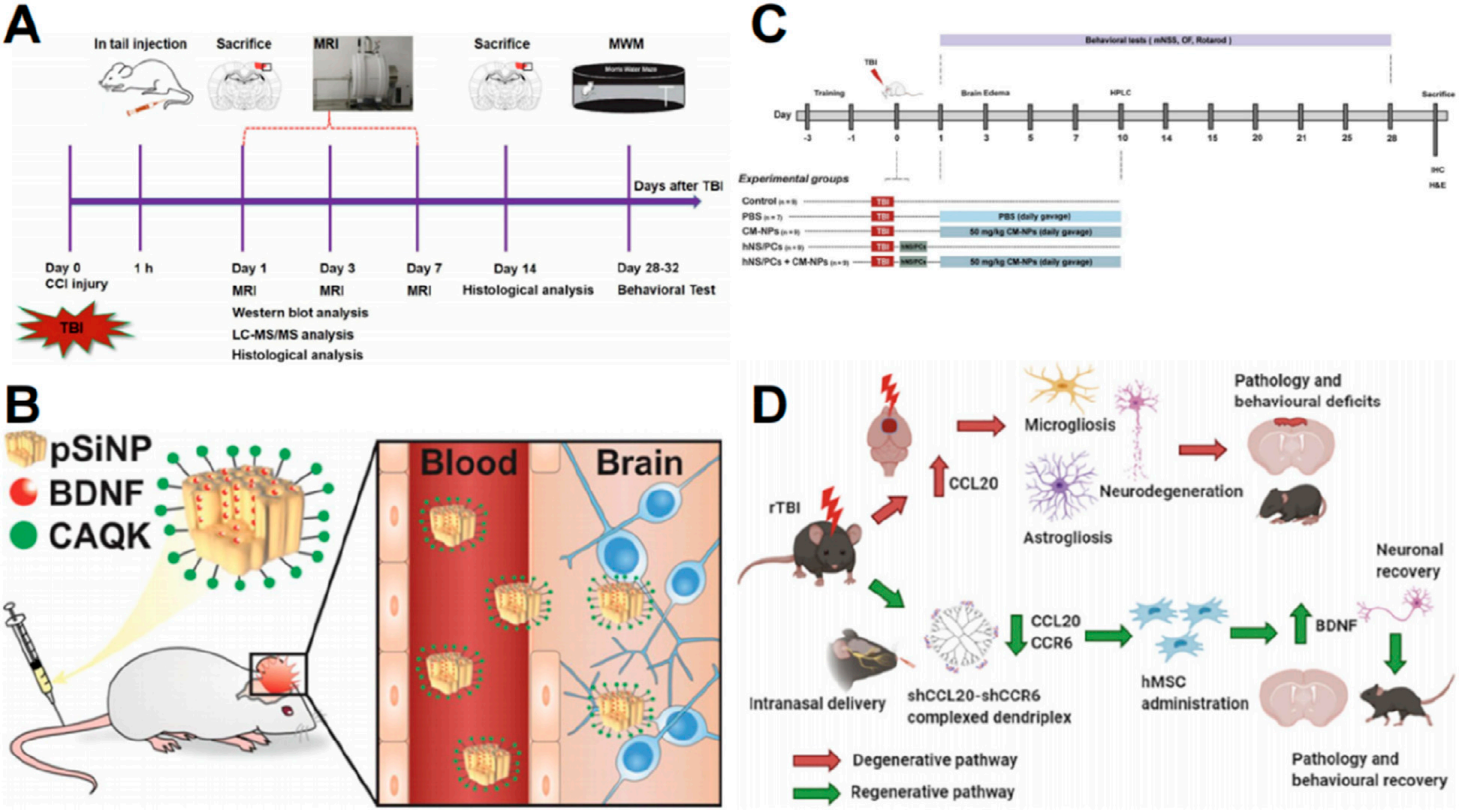
Graphic abstracts of the current nanoparticles applications in neural regeneration and stem cell therapy post traumatic brain injury. **(A)** The research design, (reprinted with permission from Zhuang et al., 2022, ©2022 Published by Elsevier Inc); **(B)** The pSiNP to load BDNF, (reprinted with permission from Waggoner et al., 2022, ©2022 American Chemical Society); **(C)** The research design, (reprinted with permission from Narouiepour et al., 2022, ©2022, The Authors); **(D)** A nanodendriplex, (reprinted with permission from Mayilsamy et al., 2020, ©2020 Elsevier Inc).

Nanotechnology has been employed to deliver stem cell-derived exosomes. Zhuang et al. (2022) developed BMSC-derived exosomes that were internalized by astrocytes and exhibited neuroprotective effects in TBI models (Figure 5A). Brain-derived neurotrophic factor (BDNF) enhances neuronal survival, neuroplasticity, and neurogenesis; however, its efficient distribution is hindered by a short half-life and instability during blood transport (Géral et al., 2013). Waggoner et al. (2022) encapsulated BDNF into biodegradable porous silicon nanoparticles to deliver bioactive BDNF to damaged brain tissue. The systemic infusion of porous silicon nanoparticles enables efficient delivery of protein cargo to the damaged brain area. This delivery system for BDNF reduced lesion volume compared to free BDNF when administered post-injury (Figure 5B) (Waggoner et al., 2022).

Nanoparticles have also been employed to deliver targeted immunomodulatory proteins to enhance stem cell therapy. Curcumin possesses anti-inflammatory and neuroprotective properties (Eghbaliferiz et al., 2020). Narouiepour et al. (2022) utilized curcumin-loaded niosome nanoparticles in combination with NSCs to promote functional recovery and reduce neuroinflammation in a TBI model by inhibiting the TLR4/NF-κB pathway (Figure 5C). CCL20 is an important chemokine that plays a role in neuroinflammation (Das et al., 2011). Mayilsamy et al. (2020) devised a novel nanocell therapy utilizing a dendrimer compound combined with a plasmid targeting CCL20 and its receptor CCR6, followed by hMSC transplantation to reduce inflammation. Notably, BDNF expression was significantly elevated, indicating potential for neurogenesis (Figure 5D) (Mayilsamy et al., 2020).

### 5.3 Nanoparticles can promote ROS scavenging post-TBI

**FIGURE 6:**
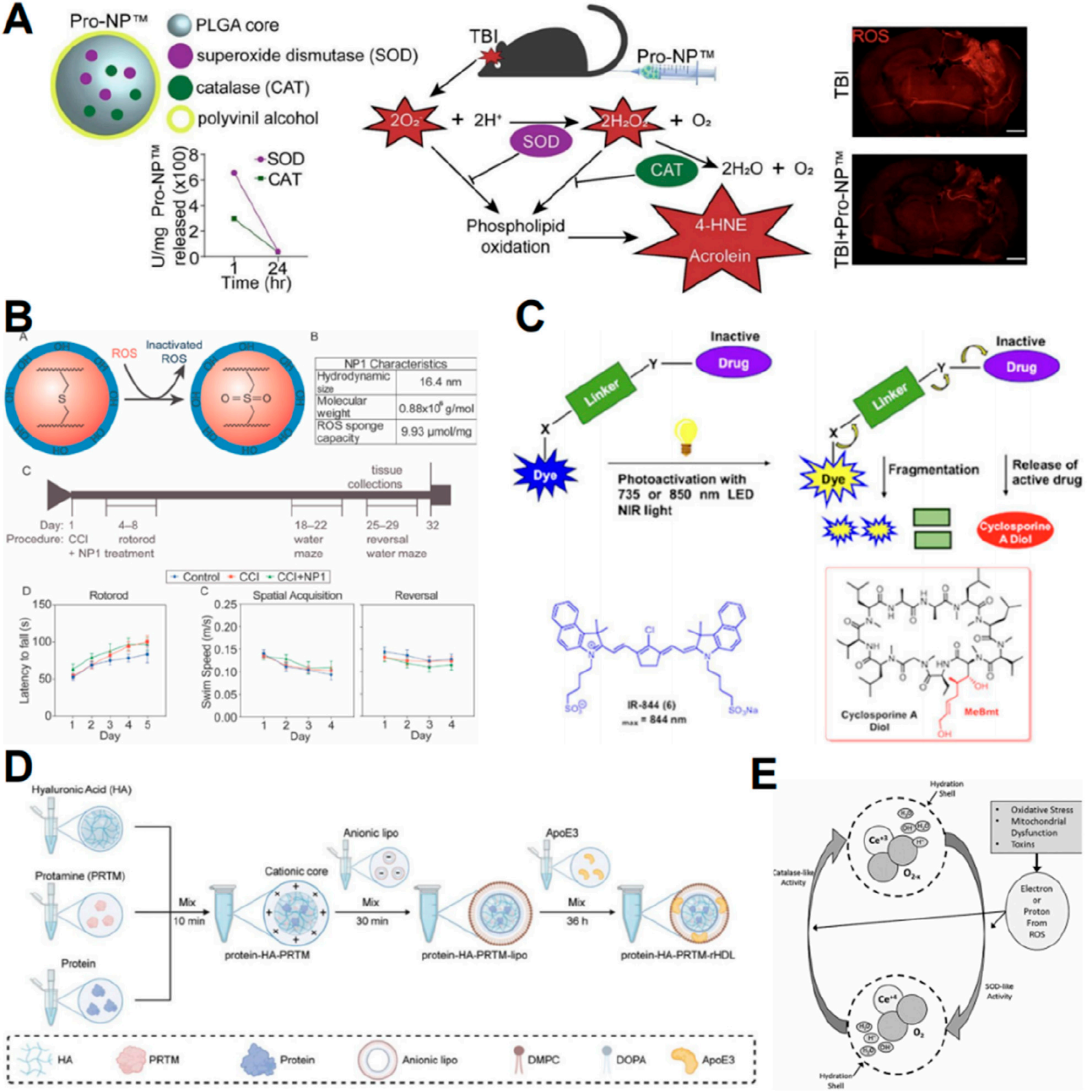
Graphic abstracts of the current nanoparticles applications in scavenging reactive oxygen species post traumatic brain injury. **(A)** The antioxidant enzyme nanoparticle, (reprinted with permission from Tarudji et al., 2023, ©2023 Elsevier B.V.); **(B)** The thioether core nanoparticle, (reprinted with permission from Tarudji et al., 2021, ©2021 Elsevier Ltd); **(C)** The cyanine nanocage to load cyclosporine A, (reprinted with permission from Black et al., 2020, ©2020 American Chemical Society); **(D)** The protein-HA-PRTM-rHDL, (reprinted with permission from Huang et al., 2024, ©2024 Wiley‐VCH GmbH); **(E)** Possible mechanism of CeONPs, (reprinted with permission from Bailey et al., 2020, ©2020, Mary Ann Liebert, Inc).

Nanoparticles with highly adjustable characteristics can be employed for drug delivery to improve accumulation in the brain (Tarudji et al., 2023). Superoxide dismutase and catalase are potent endogenous antioxidant enzymes that are rapidly cleared from the bloodstream by the kidneys and liver (Tarudji et al., 2023). However, when superoxide dismutase 1 and catalase were encapsulated in poly (lactic-co-glycolic acid)-based nanoparticles, the product showed a gradual release of the enzymes over 1 week while preserving their activity and stability (Figure 6A) (Tarudji et al., 2023). 2,2,6,6-tetramethylpiperidine-1-hydroxyl (TEMPO) can reduce ROS, but its half-life *in vivo* is very short due to rapid renal clearance (Takahashi et al., 2020). Takahashi T et al. synthesized nanoparticles containing nitrogen oxide free radicals, which extended the half-life of TEMPO and exhibited high antioxidant activity (Takahashi et al., 2020). Moreover, nanoparticles can serve as carriers for functional groups. The thioether functional group can react with hydrogen peroxide and superoxide, and Tarudji et al. (2021) utilized the antioxidant thioether core to cross-link nanoparticles, thereby mitigating reactive oxygen species in the acute phase of traumatic brain damage (Figure 6B). Cyclosporine A can mitigate lipid peroxidation (Mbye et al., 2008), but its systemic administration is associated with significant side effects (Black et al., 2020). Black et al. (2020) employed nanocages to deliver cyclosporine A, thereby minimizing systemic exposure while enhancing its pharmacological efficacy at the targeted TBI location (Figure 6C). A significant percentage of protein-based therapeutics have reduced efficacy due to their inability to traverse the BBB (Wagner et al., 2018). Motivated by the dynamic characteristics of active proteins and their natural surroundings, Huang et al. (2024) developed a biomimetic nanocavity incorporating Protein-HA-protamine-ApoE3-reconstituted high-density lipoprotein to effectively assemble various proteins for cerebral administration (Figure 6D).

Numerous inorganic materials exhibit oxidation resistance, and associated nanomaterials enhance their redox activity by increasing surface area and modifying quantum lattice structures (Celardo et al., 2011). Cerium exhibits multiple valence states and demonstrates redox activity (Celardo et al., 2011). Bailey et al. (2020) engineered cerium oxide nanoparticles (CeONP) and demonstrated their capacity to mitigate biochemical and functional consequences of mTBI (Figure 6E). In contrast, Youn et al. (2021) discovered that cerium nanorods exhibited enhanced antioxidant activity and reduced cytotoxicity compared to cerium nanospheres.

From our summary above, it can be seen that nanoparticle-based delivery systems can encapsulate a variety of therapeutic agents, and further exploration of combination therapy may provide new therapeutic possibilities.

### 5.4 Nanoparticles can facilitate anti-inflammation post-TBI

**FIGURE 7:**
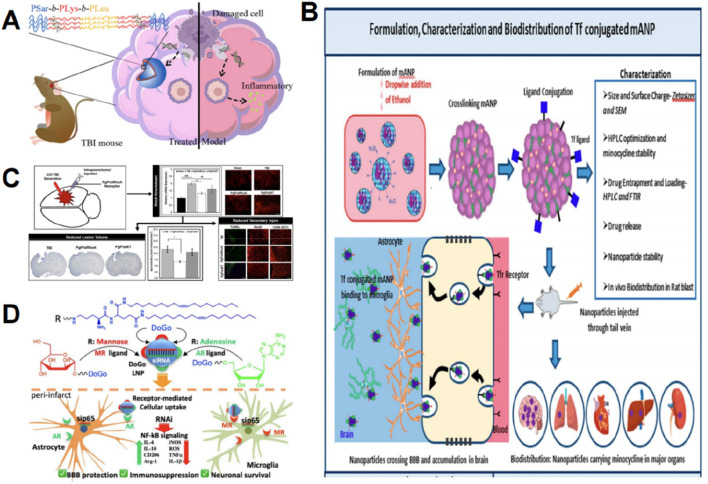
Graphic abstracts of the current nanoparticles applications in anti-inflammation therapy following traumatic brain injury. **(A)** The poly(amino acid)-based nanoparticle, (reprinted with permission from Wei et al., 2023, ©2023, American Chemical Society); **(B)** The transferrin conjugated albumin nanoparticle, (reprinted with permission from Perumal et al., 2023, ©2023 by the authors); **(C)** Effects of PgP/siRhoA nanoplex, (reprinted with permission from Macks et al., 2021, ©2020 Published by Elsevier Inc); **(D)** The dual-ligand lipid nanoparticle, (reprinted with permission from Xiao et al., 2024, ©2024, American Chemical Society).

Nanoparticles hold significant potential in modulating inflammatory responses after TBI. TBI leads to the release of cellular debris and damaged biomolecules, referred to as damage-associated molecular patterns (Corps et al., 2015), which can be detected by pattern recognition receptors (PRRs), such as Toll-like receptors (TLRs), thereby initiating inflammatory responses (Kigerl et al., 2014). Negatively charged cell-free DNAs (cfDNAs) are prevalent damage-associated molecular patterns that can be detected by TLRs, leading to inflammation and exacerbating neurodegeneration (Gögenur et al., 2017). Studies suggest that some cationic polymers and their assembled nanomaterials can neutralize negatively charged cfDNA and suppress inflammatory reactions. Consequently, Wei et al. (2023) developed poly (amino acid)-based cationic nanoparticles incorporating polysarcoline blocks as protective barriers to reduce non-specific interactions within the biological environment. These nanoparticles were administered intravenously into TBI mice to eliminate endogenous cfDNA fragments in their brains and mitigate inflammatory reactions, thereby enhancing neurological recovery (Figure 7A) (Wei et al., 2023).

The post-TBI efficacy of numerous anti-inflammatory medicines or proteins is constrained by their inability to effectively target brain lesions. Rh-erythropoietin (Rh-EPO) can diminish neuroinflammation by downregulating adhesion molecules, pro-inflammatory cytokines, microglial activation, and the NF-κB inflammatory pathway (Zhou et al., 2017). Its use is limited by its inability to directly cross the BBB; however, nanotechnology can augment drug delivery and boost drug distribution in damaged cerebral regions via the conventional BBB pathway (Xue et al., 2020). Xue et al. (2020) formulated rh-EPO encapsulated Tween 80-modified albumin nanoparticles, which enhanced the delivery of rh-EPO to the brain, significantly mitigating TBI symptoms. Minocycline is a traditional anti-inflammatory agent (Asadi et al., 2020), and nanoparticle drug delivery can address its short circulation half-life and poor bioavailability (Perumal et al., 2023). Perumal et al. (2023) developed transferrin-conjugated bovine serum albumin nanoparticles to encapsulate minocycline (Figure 7B). Transferrin can attach to receptors that are abundantly expressed in the cerebral endothelium, facilitating targeted delivery (Ulbrich et al., 2009). Albumin is readily modifiable and possesses an extended half-life (Spada et al., 2021). The findings indicated that the nanoparticles effectively transported significant amounts of minocycline to the brain and enhanced its biodistribution (Perumal et al., 2023).

Pyroptosis is a significant contributor to inflammation caused by TBI (Simon et al., 2017), and nanoparticles have been employed to suppress pyroptosis and reduce neuroinflammation. Zhang et al. (2024) developed β-lactoglobulin nanoparticles modified with cysteine-alanine-glutamine-lysine peptides for the delivery of disulfiram, which reduced brain edema and inflammation following TBI in rats, decreased secondary brain injury, and improved learning and memory recovery.

SiRNA interference can selectively regulate mRNA expression to influence inflammatory responses; however, its substantial molecular weight and negative charge necessitate a reliable delivery mechanism, making nanoparticles suitable carriers (Gherardini et al., 2014). TLR4, a component of the pattern recognition receptors, can be activated by numerous endogenous ligands following TBI and subsequently upregulate inflammatory mediators (Xiao et al., 2023). Xiao et al. (2023) developed Ad4 LNP containing siRNA directed against TLR4, administered it to a TBI mouse model, and noted a substantial reduction of TLR4 at both mRNA and protein levels in the brain, resulting in a significant decrease in key pro-inflammatory cytokines and an increase in key anti-inflammatory cytokines in serum. The upregulation of RhoA plays a crucial role in the progression of secondary injury following TBI (Macks et al., 2021). Macks et al. (2021) synthesized a novel cationic, amphiphilic copolymer, poly (lactide-glycolide copolymer)-graft-polyethylenimine (PgP), for the delivery of siRNA targeting RhoA, resulting in RhoA downregulation, thereby reducing astrogliosis and inflammation (Figure 7C). Xiao et al. (2024) revealed that dual-ligand-functionalized lipid nanoparticles (AM31 LNP) encapsulating siRNA targeting p65 resulted in significant downregulation of key pro-inflammatory cytokines, upregulation of essential anti-inflammatory cytokines, and enhanced BBB integrity (Figure 7D).

Nanoparticles can engage in anti-inflammatory processes following TBI via many methods, and nanoparticle-mediated siRNA delivery offers a precise and efficient approach to modulate specific inflammatory pathways in TBI. The inflammatory response is exceedingly intricate, involving numerous potential targets such as chemokines, endothelial cells, and leukocyte adhesion factors; thus, it fully demonstrates the promise of nano-delivery systems for anti-inflammation after TBI.

The studies mentioned above are narrated in Table 4.

**TABLE 4:** Application of nanoparticles post-TBI.

Main composition/biomaterial	In vivo models	Administration	Size (nm)	Zeta (mV)	Stimulative response	
Neural protection and regeneration						
BMSC-exosomes	Male SD rats	Intravenously	∼109.9	—	—	Zhuang et al. (2022)
CAQK modified porous silicon nanoparticles, BDNF	mice	Intravenously	∼150	−1 ± 1.5	—	Waggoner et al. (2022)
Curcumin-loaded niosome nanoparticles	Male Wistar rats	Orally	∼60	—	—	Narouiepour et al. (2022)
Polyamidoamine dendrimer, shRNA (CCL20, CCR6)	Male C57BL/6 mice	Intranasally, intravenously	100	+17	—	Mayilsamy et al. (2020)
ROS scavenging						
Poly (lactic-co-glycolic acid)-based biodegradable polymer NPs with superoxide dismutase and catalase	C57BL/6 mice	Intravenously	275–290	−10 ∼ −25	—	Tarudji et al. (2023)
Redox-active nitroxide radical–containing nanoparticles	Male ICR mice	Intravenously	∼20	5	pH-sensitive	Takahashi et al. (2020)
Antioxidant thioether core-crossed-linked NPs	C57BL/6 mice	Intravenously	∼16.4	—	—	Tarudji et al. (2021)
Cyclosporine A, cyanine Nanocage	SD rats	Intravenously	—	—	Photo-sensitive	Black et al. (2020)
Protein-hyaluronic acid-protamine- ApoE3-reconstituted high-density lipoprotein	Male C57BL/6 mice	Intravenously	26–45	−15 ∼ −55	—	Huang et al. (2024)
Cerium oxide nanoparticles	Male SD rats	Intravenously	∼10	—	—	Bailey et al. (2020)
Anti-inflammation						
Poly (amino acid)-based cationic nanoparticles	C57BL/6 mice	Intravenously	390–450	20	—	Wei et al. (2023)
Tween 80 modified albumin nanoparticles, Rh-erythropoietin	Male SD rats	Intraperitoneally	438 ± 45	−25.42 ± 0.8	—	Xue et al. (2020)
Transferrin receptor-targeted conjugated albumin nanoparticles, minocycline	Male SD rats	Intravenously	153.5 ± 3.4	−3.14 ± 3.4	—	Perumal et al. (2023)
CAQK peptide-modified β-lactoglobulin nanoparticle, disulfiram	Male ICR mice	Intravenously	156.54 ± 4.52	−28.15 ± 7.93	—	Zhang et al. (2024)
Ad4 LNP, siRNA (TLR4)	Male Balb/c mice	Intravenously	65–69	1.92–4.50	—	Xiao et al. (2023)
Poly (lactide-glycolide copolymer)-graft-polyethylenimine, siRNA (RhoA)	Male SD rats	Intracerebrally	179 ± 13.94	48.52 ± 0.35	—	Macks et al. (2021)
AM31 LNP, siRNA (p65)	Male Balb/c mice	Intravenously	65–85	−0.58 ∼ −0.29	—	Xiao et al. (2024)

BMSC, bone marrow mesenchymal stem cells; CAQK, cysteine-alanine-glutamine-lysine; SD, Sprague-Dawley; siRNA, small interfering RNA.

## 6 Discussion

**FIGURE 8:**
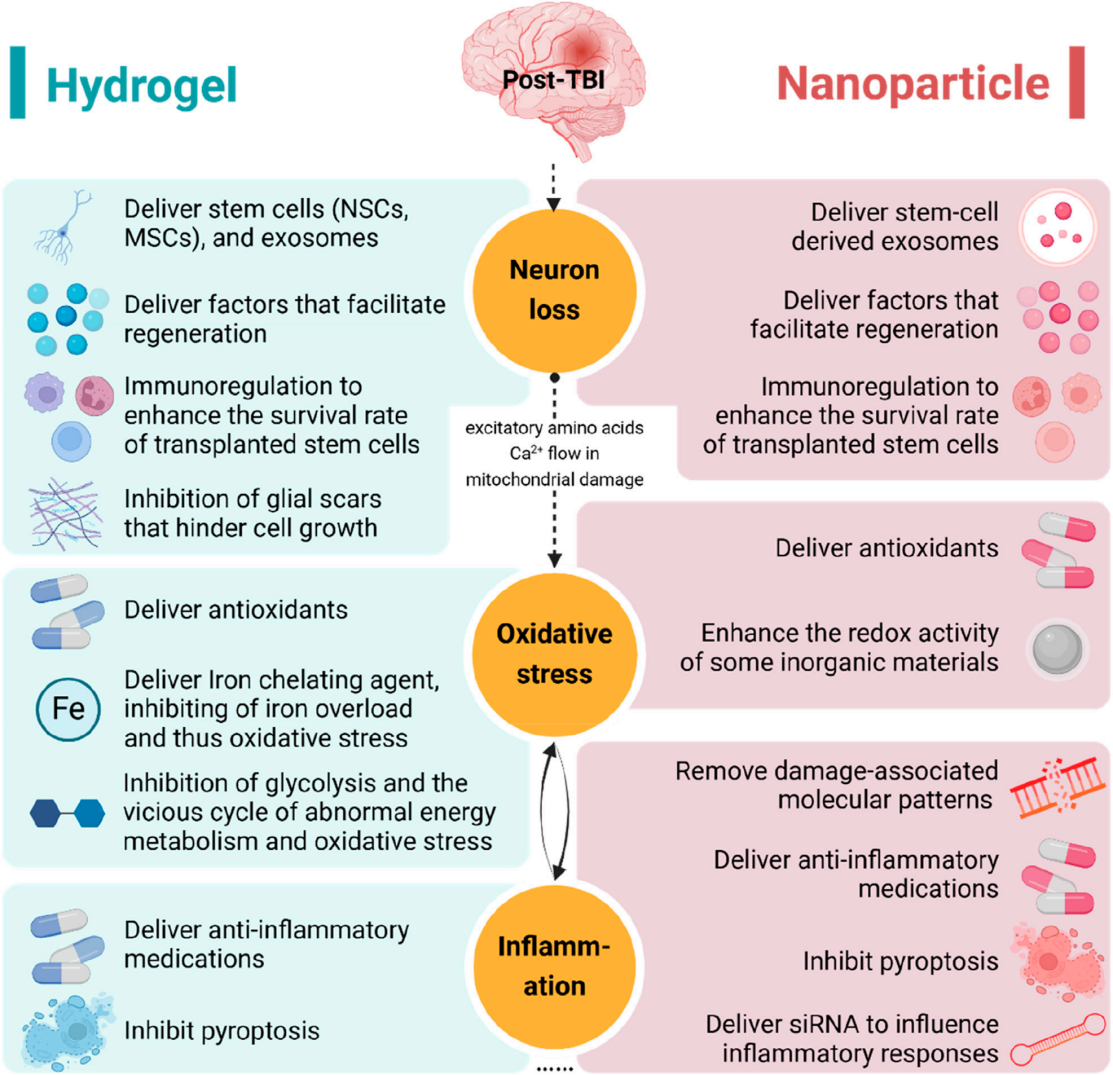
The application of hydrogels and nanoparticles targeting the pathophysiology of traumatic brain injury. NSC, neural stem cell; MSC, mesenchymal stem cell; siRNA, small interfering RNA.

In this review, we discuss the application of hydrogels and nanoparticles for the treatment of TBI, specifically targeting the pathophysiological processes involved (Figure 8).

This review illustrates the potential application of hydrogels and nanoparticles in the treatment of TBI. Hydrogels and nanoparticles can mitigate the existing issues of inadequate drug transport, insufficient accumulation, and off-target toxicity in the brain, utilizing their distinctive features to improve therapeutic efficacy (Xie and Xie, 2024; Saraiva et al., 2016). Furthermore, as an optimal delivery mechanism, they can be seamlessly integrated with various therapies to facilitate nerve regeneration, mitigate oxidative stress, reduce inflammation, and produce other benefits post-TBI, with the objective of addressing secondary injury following TBI. Among the existing investigations, novel materials integrated with cell treatments constitute a significant proportion and exhibit considerable potential. Cell therapy demonstrates considerable potential for treating TBI, with its effectiveness corroborated in clinical trials (Kawabori et al., 2021). Hydrogels and nanoparticles are expected to enhance the therapeutic effectiveness of cell therapy by improving cell outcomes. However, secondary injury following TBI encompasses additional factors, including excitotoxicity, mitochondrial dysfunction, and axonal degeneration, among others (Kaur and Sharma, 2018). Novel therapeutic strategies for these secondary injuries require urgent investigation.

Furthermore, our analysis underscores the significance of fundamental features for hydrogel and nanoparticle applications. Biocompatibility is crucial for hydrogels. The predominant hydrogels being utilized in TBI are derived from biological sources. This can be ascribed to the enhanced biocompatibility and biodegradability of bio-based scaffolds. Numerous hydrogels employing synthetic substrates are also integrated with MMP to enhance biodegradability. Moreover, as previously proven, the mechanical and rheological features of hydrogels, which might affect their performance in the brain, also impact cell destiny (Tseng et al., 2015). Furthermore, most nanoparticle diameters for TBI treatment corresponded to or resembled the optimal particle size of 20–100 nm, which is appropriate for cerebral drug transport and exhibits commendable stability. This signifies that altering the properties of these novel materials will be crucial for their efficacy in the brain.

Although basic research on these new materials is being carried out extensively, their clinical application is still limited. This may be because the utilization of biomaterials encounters obstacles. Specifically, the precise design and fabrication of hydrogels and nanoparticles to ensure stable delivery and targeted treatment remain substantial challenges (Aqel et al., 2023). Additionally, hydrogels and nanoparticles possess inherent limitations. Hydrogel swelling can exacerbate intracranial pressure, and hydrogels often require postoperative injection, which is impractical for TBI patients not undergoing brain surgery (Nih et al., 2016). The heterogeneity of natural hydrogel materials from different batches can compromise material uniformity (Aqel et al., 2023). Additionally, hydrogel materials of natural origin may introduce natural pathogens and cause unexpected inflammatory responses (Shapiro and Oyen, 2013). Nanodrugs are typically delivered via intravenous administration; however, due to the limitations imposed by the BBB, their accumulation in traumatic regions is less pronounced compared to hydrogels. Additionally, intravenously administered nanoparticles can be absorbed by tissues such as the lungs, liver, and kidneys, potentially leading to inflammation (Bartucci et al., 2020). The potential for long-term retention of non-degradable solid nanomaterials in the brain remains a concern (Alam Bony and Kievit, 2019), and the safety and toxicity of many nanomaterials have not been fully characterized (Adepu and Ramakrishna, 2021).

## 7 Conclusion

In summary, the complex pathophysiological changes following TBI, coupled with the challenges of drug delivery to the brain, render the pharmacological treatment of TBI particularly challenging. Highly tunable hydrogels and nanoparticles can be adjusted to match the properties of the brain environment, solve multiple pathophysiological problems, and achieve targeted delivery and effective drug retention, thus showing great potential in the treatment of TBI.

## 8 Future directions

Hydrogels and nanoparticles have emerged as promising drug delivery systems for TBI treatment owing to their unique physicochemical properties. Recent research indicates that the combination of hydrogels and nanoparticles facilitates flexible property adjustments, thereby broadening their range of applications (Lavrador et al., 2021). This combination incorporates the characteristics of both hydrogels and nanoparticles, resulting in complementary and enhanced performance (Suhail et al., 2019). Pure hydrogels exhibit limitations due to their polymer network structure and high water content, which result in suboptimal mechanical properties. Incorporating nanoparticles enhances mechanical strength (Esmaeely Neisiany et al., 2020). Moreover, hydrophilic hydrogels may face challenges in achieving sustained drug release (Jiang et al., 2020), while the introduction of nanoparticles can endow hydrogels with stimulus-responsive properties, enabling more complex and adjustable drug release (Lavrador et al., 2021). In contrast to pure nanoparticles, the incorporation of hydrogels improves material biocompatibility and facilitates localized delivery (Zhang et al., 2018b). Additionally, encapsulating nanoparticles within hydrogels offers protection (Jiang et al., 2020).

Overall, the combination of hydrogels and nanoparticles offers substantial advantages for medical applications. Currently, this combination has been utilized in multiple neurological disorders, such as glioblastoma (Yang et al., 2017) and spinal cord injury (Liu et al., 2023b). The properties of this combination, such as high biocompatibility, stimulus-responsive release, and enhanced mechanical support capabilities, make them highly promising for TBI treatment. Nevertheless, only a limited number of studies have investigated the application of this material for TBI treatment to date (Zheng et al., 2021; Garg et al., 2024). In the future, more studies using nanoparticles and hydrogels synergistically to promote TBI outcomes are expected.

## References

[B1] Abdul-Muneer P. M. Chandra N. Haorah J. (2015). Interactions of oxidative stress and neurovascular inflammation in the pathogenesis of traumatic brain injury. Mol. Neurobiol. 51 (3), 966–979. 10.1007/s12035-014-8752-3 24865512 PMC9420084

[B2] Adepu S. Ramakrishna S. (2021). Controlled drug delivery systems: current status and future directions. Molecules 26 (19), 5905. 10.3390/molecules26195905 34641447 PMC8512302

[B3] Ahmed J. (2023). “Chapter 20 - rheology of gelatin and advances in rheological measurements,” in Advances in Food rheology and its applications. Editors Ahmed J. Basu S. Second Edition (Woodhead Publishing), 591–636.

[B4] Akamatsu Y. Hanafy K. A. (2020). Cell death and recovery in traumatic brain injury. Neurotherapeutics 17 (2), 446–456. 10.1007/s13311-020-00840-7 32056100 PMC7283441

[B5] Alam Bony B. Kievit F. M. (2019). A role for nanoparticles in treating traumatic brain injury. Pharmaceutics 11 (9), 473. 10.3390/pharmaceutics11090473 31540234 PMC6781280

[B6] Alvarado-Velez M. Enam S. F. Mehta N. Lyon J. G. LaPlaca M. C. Bellamkonda R. V. (2021). Immuno-suppressive hydrogels enhance allogeneic MSC survival after transplantation in the injured brain. Biomaterials 266, 120419. 10.1016/j.biomaterials.2020.120419 33038594

[B7] Aqel S. Al-Thani N. Haider M. Z. Abdelhady S. Al Thani A. A. Kobeissy F. (2023). Biomaterials in traumatic brain injury: perspectives and challenges. Biol. (Basel) 13 (1), 21. 10.3390/biology13010021 PMC10813103 38248452

[B8] Asadi A. Abdi M. Kouhsari E. Panahi P. Sholeh M. Sadeghifard N. (2020). Minocycline, focus on mechanisms of resistance, antibacterial activity, and clinical effectiveness: back to the future. J. Glob. Antimicrob. Resist 22, 161–174. 10.1016/j.jgar.2020.01.022 32061815

[B9] Baby D. K. (2020). “Chapter 9 - rheology of hydrogels,” in Rheology of polymer blends and nanocomposites. Editors Thomas S. Sarathchandran C. Chandran N. (Elsevier), 193–204.

[B10] Bahadur S. Sachan N. Harwansh R. K. Deshmukh R. (2020). Nanoparticlized system: promising approach for the management of Alzheimer's disease through intranasal delivery. Curr. Pharm. Des. 26 (12), 1331–1344. 10.2174/1381612826666200311131658 32160843

[B11] Bai J. Zhang Y. Tang C. Hou Y. Ai X. Chen X. (2021). Gallic acid: pharmacological activities and molecular mechanisms involved in inflammation-related diseases. Biomed. Pharmacother. 133, 110985. 10.1016/j.biopha.2020.110985 33212373

[B12] Bailey Z. S. Nilson E. Bates J. A. Oyalowo A. Hockey K. S. Sajja V. S. S. S. (2020). Cerium oxide nanoparticles improve outcome after *in vitro* and *in vivo* mild traumatic brain injury. J. Neurotrauma 37 (12), 1452–1462. 10.1089/neu.2016.4644 27733104 PMC7249477

[B13] Banderwal R. Kadian M. Garg S. Kumar A. (2024). Comprehensive review of emerging drug targets in traumatic brain injury (TBI): challenges and future scope. Inflammopharmacology 32 (5), 3271–3293. 10.1007/s10787-024-01524-w 39023681

[B14] Bartucci R. Paramanandana A. Boersma Y. L. Olinga P. Salvati A. (2020). Comparative study of nanoparticle uptake and impact in murine lung, liver and kidney tissue slices. Nanotoxicology 14 (6), 847–865. 10.1080/17435390.2020.1771785 32536243

[B15] Bennett S. Verry C. Kaza E. Miao X. Dufort S. Boux F. (2024). Quantifying gadolinium-based nanoparticle uptake distributions in brain metastases via magnetic resonance imaging. Sci. Rep. 14 (1), 11959. 10.1038/s41598-024-62389-1 38796495 PMC11128019

[B16] Bharadwaj V. N. Lifshitz J. Adelson P. D. Kodibagkar V. D. Stabenfeldt S. E. (2016). Temporal assessment of nanoparticle accumulation after experimental brain injury: effect of particle size. Sci. Rep. 6, 29988. 10.1038/srep29988 27444615 PMC4957235

[B17] Bharadwaj V. N. Nguyen D. T. Kodibagkar V. D. Stabenfeldt S. E. (2018). Nanoparticle-based therapeutics for brain injury. Adv. Healthc. Mater 7 (1). 10.1002/adhm.201700668 PMC5903677 29034608

[B18] Black C. E. Zhou E. DeAngelo C. Asante I. Yang R. Petasis N. A. (2020). Cyanine nanocage activated by near-IR light for the targeted delivery of cyclosporine A to traumatic brain injury sites. Mol. Pharm. 17 (12), 4499–4509. 10.1021/acs.molpharmaceut.0c00589 32813533

[B19] Budai L. Budai M. Fülöpné Pápay Z. E. Vilimi Z. Antal I. (2023). Rheological considerations of pharmaceutical formulations: focus on viscoelasticity. Gels 9 (6), 469. 10.3390/gels9060469 37367140 PMC10298452

[B20] Cao H. Duan L. Zhang Y. Cao J. Zhang K. (2021). Current hydrogel advances in physicochemical and biological response-driven biomedical application diversity. Signal Transduct. Target Ther. 6 (1), 426. 10.1038/s41392-021-00830-x 34916490 PMC8674418

[B21] Cash A. Theus M. H. (2020). Mechanisms of blood-brain barrier dysfunction in traumatic brain injury. Int. J. Mol. Sci. 21 (9), 3344. 10.3390/ijms21093344 32397302 PMC7246537

[B22] Catoira M. C. Fusaro L. Di Francesco D. Ramella M. Boccafoschi F. (2019). Overview of natural hydrogels for regenerative medicine applications. J. Mater Sci. Mater Med. 30 (10), 115. 10.1007/s10856-019-6318-7 31599365 PMC6787111

[B23] Celardo I. Pedersen J. Z. Traversa E. Ghibelli L. (2011). Pharmacological potential of cerium oxide nanoparticles. Nanoscale 3 (4), 1411–1420. 10.1039/c0nr00875c 21369578

[B24] Chen M. H. Wang L. L. Chung J. J. Kim Y. H. Atluri P. Burdick J. A. (2017). Methods to assess shear-thinning hydrogels for application as injectable biomaterials. ACS Biomater. Sci. Eng. 3 (12), 3146–3160. 10.1021/acsbiomaterials.7b00734 29250593 PMC5727472

[B25] Chen T. Xia Y. Zhang L. Xu T. Yi Y. Chen J. (2023). Loading neural stem cells on hydrogel scaffold improves cell retention rate and promotes functional recovery in traumatic brain injury. Mater Today Bio 19, 100606. 10.1016/j.mtbio.2023.100606 PMC10102240 37063247

[B26] Chen X. Huang X. Liu C. Li S. Yang Z. Zhang F. (2022). Surface-fill H(2)S-releasing silk fibroin hydrogel for brain repair through the repression of neuronal pyroptosis. Acta Biomater. 154, 259–274. 10.1016/j.actbio.2022.11.021 36402296

[B27] Chernos M. Grecov D. Kwok E. Bebe S. Babsola O. Anastassiades T. (2017). Rheological study of hyaluronic acid derivatives. Biomed. Eng. Lett. 7 (1), 17–24. 10.1007/s13534-017-0010-y 30603147 PMC6208462

[B28] Corps K. N. Roth T. L. McGavern D. B. (2015). Inflammation and neuroprotection in traumatic brain injury. JAMA Neurol. 72 (3), 355–362. 10.1001/jamaneurol.2014.3558 25599342 PMC5001842

[B29] Coyne T. M. Marcus A. J. Woodbury D. Black I. B. (2006). Marrow stromal cells transplanted to the adult brain are rejected by an inflammatory response and transfer donor labels to host neurons and glia. Stem Cells 24 (11), 2483–2492. 10.1634/stemcells.2006-0174 16873764

[B30] Cuomo F. Cofelice M. Lopez F. (2019). Rheological characterization of hydrogels from alginate-based nanodispersion. Polym. (Basel) 11 (2), 259. 10.3390/polym11020259 PMC6419013 30960242

[B31] Das M. Leonardo C. C. Rangooni S. Pennypacker K. R. Mohapatra S. Mohapatra S. S. (2011). Lateral fluid percussion injury of the brain induces CCL20 inflammatory chemokine expression in rats. J. Neuroinflammation 8, 148. 10.1186/1742-2094-8-148 22040257 PMC3231817

[B32] Davis C. K. Arruri V. Joshi P. Vemuganti R. (2024). Non-pharmacological interventions for traumatic brain injury. J. Cereb. Blood Flow. Metab. 44 (5), 641–659. 10.1177/0271678x241234770 38388365 PMC11197135

[B33] Dekmak A. Mantash S. Shaito A. Toutonji A. Ramadan N. Ghazale H. (2018). Stem cells and combination therapy for the treatment of traumatic brain injury. Behav. Brain Res. 340, 49–62. 10.1016/j.bbr.2016.12.039 28043902

[B34] Della Sala F. Biondi M. Guarnieri D. Borzacchiello A. Ambrosio L. Mayol L. (2020). Mechanical behavior of bioactive poly(ethylene glycol) diacrylate matrices for biomedical application. J. Mech. Behav. Biomed. Mater 110, 103885. 10.1016/j.jmbbm.2020.103885 32957192

[B35] Diaz M. D. Kandell R. M. Wu J. R. Chen A. Christman K. L. Kwon E. J. (2023). Infusible extracellular matrix biomaterial promotes vascular integrity and modulates the inflammatory response in acute traumatic brain injury. Adv. Healthc. Mater 12 (25), e2300782. 10.1002/adhm.202300782 37390094 PMC10592293

[B36] Dong Z. Zhao J. Xu J. Deng W. Sun P. (2024). Strongly adhesive, self-healing, hemostatic hydrogel for the repair of traumatic brain injury. Biomacromolecules 25 (4), 2462–2475. 10.1021/acs.biomac.3c01406 38533630

[B37] Dousset V. Brochet B. Deloire M. S. A. Lagoarde L. Barroso B. Caille J. M. (2006). MR imaging of relapsing multiple sclerosis patients using ultra-small-particle iron oxide and compared with gadolinium. AJNR Am. J. Neuroradiol. 27 (5), 1000–1005. 10.1016/j.acra.2006.01.051 16687532 PMC7975759

[B38] Eghbaliferiz S. Farhadi F. Barreto G. E. Majeed M. Sahebkar A. (2020). Effects of curcumin on neurological diseases: focus on astrocytes. Pharmacol. Rep. 72 (4), 769–782. 10.1007/s43440-020-00112-3 32458309

[B39] Esmaeely Neisiany R. Enayati M. S. Sajkiewicz P. Pahlevanneshan Z. Ramakrishna S. (2020). Insight into the current directions in functionalized nanocomposite hydrogels. Front. Mater. 7, 25. 10.3389/fmats.2020.00025

[B40] Fesharaki-Zadeh A. (2022). Oxidative stress in traumatic brain injury. Int. J. Mol. Sci. 23 (21), 13000. 10.3390/ijms232113000 36361792 PMC9657447

[B41] Formica M. L. Real D. A. Picchio M. L. Catlin E. Donnelly R. F. Paredes A. J. (2022). On a highway to the brain: a review on nose-to-brain drug delivery using nanoparticles. Appl. Mater. Today 29, 101631. 10.1016/j.apmt.2022.101631

[B42] Garg S. Jana A. Khan J. Gupta S. Roy R. Gupta V. (2024). Logic “AND gate circuit” based mussel inspired polydopamine nanocomposite as bioactive antioxidant for management of oxidative stress and neurogenesis in traumatic brain injury. ACS Appl. Mater Interfaces 16 (28), 36168–36193. 10.1021/acsami.4c07694 38954488

[B43] Gefen A. Margulies S. S. (2004). Are *in vivo* and *in situ* brain tissues mechanically similar? J. Biomech. 37 (9), 1339–1352. 10.1016/j.jbiomech.2003.12.032 15275841

[B44] Géral C. Angelova A. Lesieur S. (2013). From molecular to nanotechnology strategies for delivery of neurotrophins: emphasis on brain-derived neurotrophic factor (BDNF). Pharmaceutics 5 (1), 127–167. 10.3390/pharmaceutics5010127 24300402 PMC3834942

[B45] Gherardini L. Bardi G. Gennaro M. Pizzorusso T. (2014). Novel siRNA delivery strategy: a new “strand” in CNS translational medicine? Cell Mol. Life Sci. 71 (1), 1–20. 10.1007/s00018-013-1310-8 23508806 PMC11113879

[B46] Gögenur M. Burcharth J. Gögenur I. (2017). The role of total cell-free DNA in predicting outcomes among trauma patients in the intensive care unit: a systematic review. Crit. Care 21 (1), 14. 10.1186/s13054-016-1578-9 28118843 PMC5260039

[B47] Green A. L. Arnaud A. Batiller J. Eljamel S. Gauld J. Jones P. (2015). A multicentre, prospective, randomized, controlled study to evaluate the use of a fibrin sealant as an adjunct to sutured dural repair. Br. J. Neurosurg. 29 (1), 11–17. 10.3109/02688697.2014.948808 25112563

[B48] Gupta M. K. Martin J. R. Werfel T. A. Shen T. Page J. M. Duvall C. L. (2014). Cell protective, ABC triblock polymer-based thermoresponsive hydrogels with ROS-triggered degradation and drug release. J. Am. Chem. Soc. 136 (42), 14896–14902. 10.1021/ja507626y 25254509

[B49] Han Y. Han Z. Huang X. Li S. Jin G. Feng J. (2024c). An injectable refrigerated hydrogel for inducing local hypothermia and neuroprotection against traumatic brain injury in mice. J. Nanobiotechnology 22 (1), 251. 10.1186/s12951-024-02454-z 38750597 PMC11095020

[B50] Han Y. Weng W. Zhang Y. Feng Q. Ma Y. Quan A. (2024a). Intraoperative application of intelligent, responsive, self-assembling hydrogel rectifies oxygen and energy metabolism in traumatically injured brain. Biomaterials 306, 122495. 10.1016/j.biomaterials.2024.122495 38309053

[B51] Han Z. Zhao Z. Yu H. Wang L. Yue C. Zhu B. (2024b). Microenvironment-responsive hydrogel reduces seizures after traumatic brain injury in juvenile rats by reducing oxidative stress and hippocampal inflammation. Macromol. Biosci. 24 (8), e2400050. 10.1002/mabi.202400050 38810210

[B52] Holden P. Nair L. S. (2019). Deferoxamine: an angiogenic and antioxidant molecule for tissue regeneration. Tissue Eng. Part B Rev. 25 (6), 461–470. 10.1089/ten.teb.2019.0111 31184273

[B53] Honary S. Zahir F. (2013). Effect of zeta potential on the properties of nano-drug delivery systems - a review (Part 2). Trop. J. Pharm. Res. 12, 12. 10.4314/tjpr.v12i2.20

[B54] Hu Y. Jia Y. Wang S. Ma Y. Huang G. Ding T. (2023). An ECM-mimicking, injectable, viscoelastic hydrogel for treatment of brain lesions. Adv. Healthc. Mater 12 (1), e2201594. 10.1002/adhm.202201594 36398536

[B55] Huang J. Fu Y. Wang A. shi K. Peng Y. Yi Y. (2024). Brain delivery of protein therapeutics by cell matrix-inspired biomimetic nanocarrier. Adv. Mater 36 (31), e2405323. 10.1002/adma.202405323 38718295

[B56] Huang X. Ye Y. Zhang J. Zhang X. Ma H. Zhang Y. (2022). Reactive oxygen species scavenging functional hydrogel delivers procyanidins for the treatment of traumatic brain injury in mice. ACS Appl. Mater Interfaces 14, 33756–33767. 10.1021/acsami.2c04930 35833273

[B57] Jamjoom A. A. B. Rhodes J. Andrews P. J. D. Grant S. G. N. (2021). The synapse in traumatic brain injury. Brain 144 (1), 18–31. 10.1093/brain/awaa321 33186462 PMC7880663

[B58] Javalgekar M. Jupp B. Vivash L. O’Brien T. J. Wright D. K. Jones N. C. (2024). Inflammasomes at the crossroads of traumatic brain injury and post-traumatic epilepsy. J. Neuroinflammation 21 (1), 172. 10.1186/s12974-024-03167-8 39014496 PMC11250980

[B59] Javed M. Saleem A. Xaveria A. Akhtar M. F. (2022). Daphnetin: a bioactive natural coumarin with diverse therapeutic potentials. Front. Pharmacol. 13, 993562. 10.3389/fphar.2022.993562 36249766 PMC9556945

[B60] Jiang Y. Krishnan N. Heo J. Fang R. H. Zhang L. (2020). Nanoparticle-hydrogel superstructures for biomedical applications. J. Control Release 324, 505–521. 10.1016/j.jconrel.2020.05.041 32464152 PMC7429280

[B61] Jing L. Fan S. Yao X. Zhang Y. (2021). Effects of compound stimulation of fluid shear stress plus ultrasound on stem cell proliferation and osteogenesis. Regen. Biomater. 8 (6), rbab066. 10.1093/rb/rbab066 34868635 PMC8634505

[B62] Jones C. Elliott B. Liao Z. Johnson Z. Ma F. Bailey Z. S. (2023). PEG hydrogel containing dexamethasone-conjugated hyaluronic acid reduces secondary injury and improves motor function in a rat moderate TBI model. Exp. Neurol. 369, 114533. 10.1016/j.expneurol.2023.114533 37666386

[B63] Jullienne A. Obenaus A. Ichkova A. Savona‐Baron C. Pearce W. J. Badaut J. (2016). Chronic cerebrovascular dysfunction after traumatic brain injury. J. Neurosci. Res. 94 (7), 609–622. 10.1002/jnr.23732 27117494 PMC5415378

[B64] Kariminekoo S. Movassaghpour A. Rahimzadeh A. Talebi M. Shamsasenjan K. Akbarzadeh A. (2016). Implications of mesenchymal stem cells in regenerative medicine. Artif. Cells Nanomed Biotechnol. 44 (3), 749–757. 10.3109/21691401.2015.1129620 26757594

[B65] Karvinen J. Kellomäki M. (2022). Characterization of self-healing hydrogels for biomedical applications. Eur. Polym. J. 181, 111641. 10.1016/j.eurpolymj.2022.111641

[B66] Kaur P. Sharma S. (2018). Recent advances in pathophysiology of traumatic brain injury. Curr. Neuropharmacol. 16 (8), 1224–1238. 10.2174/1570159x15666170613083606 28606040 PMC6142406

[B67] Kawabori M. Weintraub A. H. Imai H. Zinkevych I. McAllister P. Steinberg G. K. (2021). Cell therapy for chronic TBI: interim analysis of the randomized controlled STEMTRA trial. Neurology 96 (8), e1202–e1214. 10.1212/wnl.0000000000011450 33397772 PMC8055341

[B68] Khaing Z. Z. Seidlits S. K. (2015). Hyaluronic acid and neural stem cells: implications for biomaterial design. J. Mater Chem. B 3 (40), 7850–7866. 10.1039/c5tb00974j 32262899

[B69] Khan S. Amin F. M. Fliedner F. P. Christensen C. E. Tolnai D. Younis S. (2019). Investigating macrophage-mediated inflammation in migraine using ultrasmall superparamagnetic iron oxide-enhanced 3T magnetic resonance imaging. Cephalalgia 39 (11), 1407–1420. 10.1177/0333102419848122 31104505

[B70] Khatri N. Thakur M. Pareek V. Kumar S. Sharma S. Datusalia A. K. (2018). Oxidative stress: major threat in traumatic brain injury. CNS Neurol. Disord. Drug Targets 17 (9), 689–695. 10.2174/1871527317666180627120501 29952272

[B71] Kigerl K. A. de Rivero Vaccari J. P. Dietrich W. D. Popovich P. G. Keane R. W. (2014). Pattern recognition receptors and central nervous system repair. Exp. Neurol. 258, 5–16. 10.1016/j.expneurol.2014.01.001 25017883 PMC4974939

[B72] Killion J. Geever L. M. Devine D. M. Grehan L. Kennedy J. E. Higginbotham C. L. (2012). Modulating the mechanical properties of photopolymerised polyethylene glycol-polypropylene glycol hydrogels for bone regeneration. J. Mater. Sci. 47, 6577–6585. 10.1007/s10853-012-6588-7

[B73] Kim B. S. Kim J. U. Lee J. Ryu K. M. Kim S. H. Hwang N. S. (2024). Decellularized brain extracellular matrix based NGF-releasing cryogel for brain tissue engineering in traumatic brain injury. J. Control Release 368, 140–156. 10.1016/j.jconrel.2024.02.017 38373473

[B74] Kim J. T. Cho S. M. Youn D. H. Hong E. P. Park C. H. Lee Y. (2023). Therapeutic effect of a hydrogel-based neural stem cell delivery sheet for mild traumatic brain injury. Acta Biomater. 167, 335–347. 10.1016/j.actbio.2023.06.027 37356785

[B75] Kumar V. A. Taylor N. L. Shi S. Wang B. K. Jalan A. A. Kang M. K. (2015). Highly angiogenic peptide nanofibers. ACS Nano 9 (1), 860–868. 10.1021/nn506544b 25584521 PMC4370274

[B76] Lainé A. Brot S. Gaillard A. (2022). Beneficial effects of hyaluronan-based hydrogel implantation after cortical traumatic injury. Cells 11 (23), 3831. 10.3390/cells11233831 36497093 PMC9735891

[B77] Lamade A. M. Kenny E. M. Anthonymuthu T. S. Soysal E. Clark R. S. Kagan V. E. (2019). Aiming for the target: mitochondrial drug delivery in traumatic brain injury. Neuropharmacology 145 (Pt B), 209–219. 10.1016/j.neuropharm.2018.07.014 30009835 PMC6309489

[B78] Lavrador P. Esteves M. R. Gaspar V. M. Mano J. F. (2021). Stimuli-responsive nanocomposite hydrogels for biomedical applications. Adv. Funct. Mater. 31 (8), 2005941. 10.1002/adfm.202005941

[B79] Lee D. Zhang H. Ryu S. (2019b). Elastic modulus measurement of hydrogels. Polym. Polym. Compos. A Reference Ser., 865–884. 10.1007/978-3-319-77830-3_60

[B80] Lee H. J. Ryu J. S. Vig P. J. (2019a). Current strategies for therapeutic drug delivery after traumatic CNS injury. Ther. Deliv. 10 (4), 251–263. 10.4155/tde-2019-0006 30991923

[B81] Lewis S. R. Evans D. J. Butler A. R. Schofield-Robinson O. J. Alderson P. (2017). Hypothermia for traumatic brain injury. Cochrane Database Syst. Rev. 9 (9), Cd001048. 10.1002/14651858.cd001048.pub5 28933514 PMC6483736

[B82] Li J. Zhang D. Guo S. Zhao C. Wang L. Ma S. (2021). Dual-enzymatically cross-linked gelatin hydrogel promotes neural differentiation and neurotrophin secretion of bone marrow-derived mesenchymal stem cells for treatment of moderate traumatic brain injury. Int. J. Biol. Macromol. 187, 200–213. 10.1016/j.ijbiomac.2021.07.111 34310990

[B83] Lian J. Mansel B. W. Ingham B. Prabakar S. Williams M. A. K. (2016). Controlling chain flexibility in collagen networks to produce hydrogels with distinct properties. Soft Mater. 15, 145–152. 10.1080/1539445x.2016.1268626

[B84] Lin C. M. Lin J. W. Chen Y. C. Shen H. H. Wei L. Yeh Y. S. (2009). Hyaluronic acid inhibits the glial scar formation after brain damage with tissue loss in rats. Surg. Neurol. 72 (Suppl. 2), S50–S54. 10.1016/j.wneu.2009.09.004 19944826

[B85] Liu D. Lu G. Shi B. Ni H. Wang J. Qiu Y. (2023b). ROS-scavenging hydrogels synergize with neural stem cells to enhance spinal cord injury repair via regulating microenvironment and facilitating nerve regeneration. Adv. Healthc. Mater 12 (18), e2300123. 10.1002/adhm.202300123 36989238

[B86] Liu X. Wu C. Zhang Y. Chen S. Ding J. Chen Z. (2023a). Hyaluronan-based hydrogel integrating exosomes for traumatic brain injury repair by promoting angiogenesis and neurogenesis. Carbohydr. Polym. 306, 120578. 10.1016/j.carbpol.2023.120578 36746568

[B87] Liu Y. Hsu S. H. (2020). Biomaterials and neural regeneration. Neural Regen. Res. 15 (7), 1243–1244. 10.4103/1673-5374.272573 31960803 PMC7047791

[B88] Liu Y. Hsu Y. H. Huang A. P. H. Hsu S. h. (2020). Semi-interpenetrating polymer network of hyaluronan and chitosan self-healing hydrogels for central nervous system repair. ACS Appl. Mater Interfaces 12 (36), 40108–40120. 10.1021/acsami.0c11433 32808527

[B89] Lockman P. R. Koziara J. M. Mumper R. J. Allen D. D. (2004). Nanoparticle surface charges alter blood-brain barrier integrity and permeability. J. Drug Target 12 (9-10), 635–641. 10.1080/10611860400015936 15621689

[B90] Loebel C. Rodell C. B. Chen M. H. Burdick J. A. (2017). Shear-thinning and self-healing hydrogels as injectable therapeutics and for 3D-printing. Nat. Protoc. 12 (8), 1521–1541. 10.1038/nprot.2017.053 28683063 PMC7546336

[B91] Lucke-Wold B. P. Logsdon A. F. Nguyen L. Eltanahay A. Turner R. C. Bonasso P. (2018). Supplements, nutrition, and alternative therapies for the treatment of traumatic brain injury. Nutr. Neurosci. 21 (2), 79–91. 10.1080/1028415x.2016.1236174 27705610 PMC5491366

[B92] Luo Z. Wang Y. Xu Y. Wang J. Yu Y. (2023). Modification and crosslinking strategies for hyaluronic acid-based hydrogel biomaterials. Smart Med. 2 (4), e20230029. 10.1002/smmd.20230029 39188300 PMC11235888

[B93] Ma S. Zhou J. Huang T. Zhang Z. Xing Q. Zhou X. (2021). Sodium alginate/collagen/stromal cell-derived factor-1 neural scaffold loaded with BMSCs promotes neurological function recovery after traumatic brain injury. Acta Biomater. 131, 185–197. 10.1016/j.actbio.2021.06.038 34217903

[B94] Ma X. Agas A. Siddiqui Z. Kim K. Iglesias-Montoro P. Kalluru J. (2020). Angiogenic peptide hydrogels for treatment of traumatic brain injury. Bioact. Mater 5 (1), 124–132. 10.1016/j.bioactmat.2020.01.005 32128463 PMC7042674

[B95] Ma Y. Liu Y. Guo J. Chen Z. Zhao Z. Zheng J. (2024). Topical application of daphnetin hydrogel for traumatic brain injury. Front. Neurosci. 18, 1450072. 10.3389/fnins.2024.1450072 39170676 PMC11335657

[B96] Maas A. I. R. Menon D. K. Manley G. T. Abrams M. Åkerlund C. Andelic N. (2022). Traumatic brain injury: progress and challenges in prevention, clinical care, and research. Lancet Neurol. 21 (11), 1004–1060. 10.1016/s1474-4422(22)00309-x 36183712 PMC10427240

[B97] Macks C. Jeong D. Bae S. Webb K. Lee J. S. (2022). Dexamethasone-loaded hydrogels improve motor and cognitive functions in a rat mild traumatic brain injury model. Int. J. Mol. Sci. 23 (19), 11153. 10.3390/ijms231911153 36232454 PMC9570348

[B98] Macks C. Jeong D. Lee J. S. (2021). Local delivery of RhoA siRNA by PgP nanocarrier reduces inflammatory response and improves neuronal cell survival in a rat TBI model. Nanomedicine 32, 102343. 10.1016/j.nano.2020.102343 33259960 PMC8714129

[B99] Marcello E. Chiono V. (2023). Biomaterials-enhanced intranasal delivery of drugs as a direct route for brain targeting. Int. J. Mol. Sci. 24 (4), 3390. 10.3390/ijms24043390 36834804 PMC9964911

[B100] Martín Giménez V. M. Arya G. Zucchi I. A. Galante M. J. Manucha W. (2021). Photo-responsive polymeric nanocarriers for target-specific and controlled drug delivery. Soft Matter 17 (38), 8577–8584. 10.1039/d1sm00999k 34580698

[B101] Mayilsamy K. Markoutsa E. Das M. Chopade P. Puro D. Kumar A. (2020). Treatment with shCCL20-CCR6 nanodendriplexes and human mesenchymal stem cell therapy improves pathology in mice with repeated traumatic brain injury. Nanomedicine 29, 102247. 10.1016/j.nano.2020.102247 32599163

[B102] Mbye L. H. Singh I. Sullivan P. Springer J. Hall E. (2008). Attenuation of acute mitochondrial dysfunction after traumatic brain injury in mice by NIM811, a non-immunosuppressive cyclosporin A analog. Exp. Neurol. 209 (1), 243–253. 10.1016/j.expneurol.2007.09.025 18022160

[B103] Mira R. G. Lira M. Cerpa W. (2021). Traumatic brain injury: mechanisms of glial response. Front. Physiol. 12, 740939. 10.3389/fphys.2021.740939 34744783 PMC8569708

[B104] Mishchenko T. A. Klimenko M. O. Kuznetsova A. I. Yarkov R. S. Savelyev A. G. Sochilina A. V. (2022). 3D-printed hyaluronic acid hydrogel scaffolds impregnated with neurotrophic factors (BDNF, GDNF) for post-traumatic brain tissue reconstruction. Front. Bioeng. Biotechnol. 10, 895406. 10.3389/fbioe.2022.895406 36091441 PMC9453866

[B105] Nance E. Pun S. H. Saigal R. Sellers D. L. (2022). Drug delivery to the central nervous system. Nat. Rev. Mater 7 (4), 314–331. 10.1038/s41578-021-00394-w 38464996 PMC10923597

[B106] Narouiepour A. Ebrahimzadeh-bideskan A. Rajabzadeh G. Gorji A. Negah S. S. (2022). Neural stem cell therapy in conjunction with curcumin loaded in niosomal nanoparticles enhanced recovery from traumatic brain injury. Sci. Rep. 12 (1), 3572. 10.1038/s41598-022-07367-1 35246564 PMC8897489

[B107] Ng S. Y. Lee A. Y. W. (2019). Traumatic brain injuries: pathophysiology and potential therapeutic targets. Front. Cell Neurosci. 13, 528. 10.3389/fncel.2019.00528 31827423 PMC6890857

[B108] Nih L. R. Carmichael S. T. Segura T. (2016). Hydrogels for brain repair after stroke: an emerging treatment option. Curr. Opin. Biotechnol. 40, 155–163. 10.1016/j.copbio.2016.04.021 27162093 PMC4975623

[B109] Orr T. J. Lesha E. Kramer A. H. Cecia A. Dugan J. E. Schwartz B. (2024). Traumatic brain injury: a comprehensive review of biomechanics and molecular pathophysiology. World Neurosurg. 185, 74–88. 10.1016/j.wneu.2024.01.084 38272305

[B110] Osbun J. W. Ellenbogen R. G. Chesnut R. M. Chin L. S. Connolly P. J. Cosgrove G. R. (2012). A multicenter, single-blind, prospective randomized trial to evaluate the safety of a polyethylene glycol hydrogel (Duraseal Dural Sealant System) as a dural sealant in cranial surgery. World Neurosurg. 78 (5), 498–504. 10.1016/j.wneu.2011.12.011 22381303

[B111] Öztürk K. Kaplan M. Çalış S. (2024). Effects of nanoparticle size, shape, and zeta potential on drug delivery. Int. J. Pharm. 666, 124799. 10.1016/j.ijpharm.2024.124799 39369767

[B112] Pajak B. Siwiak E. Sołtyka M. Priebe A. Zieliński R. Fokt I. (2019). 2-Deoxy-d-Glucose and its analogs: from diagnostic to therapeutic agents. Int. J. Mol. Sci. 21 (1), 234. 10.3390/ijms21010234 31905745 PMC6982256

[B113] Pardridge W. M. (2011). Drug transport in brain via the cerebrospinal fluid. Fluids Barriers CNS 8 (1), 7. 10.1186/2045-8118-8-7 21349155 PMC3042981

[B114] Patet C. Suys T. Carteron L. Oddo M. (2016). Cerebral lactate metabolism after traumatic brain injury. Curr. Neurol. Neurosci. Rep. 16 (4), 31. 10.1007/s11910-016-0638-5 26898683

[B115] Perumal V. Ravula A. R. Agas A. Gosain A. Aravind A. Sivakumar P. M. (2023). Enhanced targeted delivery of minocycline via transferrin conjugated albumin nanoparticle improves neuroprotection in a blast traumatic brain injury model. Brain Sci. 13 (3), 402. 10.3390/brainsci13030402 36979212 PMC10046830

[B116] Petersen A. B. Tøndervik A. Gaardløs M. Ertesvåg H. Sletta H. Aachmann F. (2023). Mannuronate C-5 epimerases and their use in alginate modification. Essays Biochem. 67 (3), 615–627. 10.1042/ebc20220151 36876890

[B117] Pulgar V. M. (2018). Transcytosis to cross the blood brain barrier, new advancements and challenges. Front. Neurosci. 12, 1019. 10.3389/fnins.2018.01019 30686985 PMC6337067

[B118] Qian F. Han Y. Han Z. Zhang D. Zhang L. Zhao G. (2021). *In situ* implantable, post-trauma microenvironment-responsive, ROS Depletion Hydrogels for the treatment of Traumatic brain injury. Biomaterials 270, 120675. 10.1016/j.biomaterials.2021.120675 33548799

[B119] Qiu Y. Zeng Y. Zhang C. Lv X. Ling Y. Si Y. (2024). A ROS-responsive loaded desferoxamine (DFO) hydrogel system for traumatic brain injury therapy. Biomed. Mater 19 (2), 025016. 10.1088/1748-605x/ad1dfd 38215474

[B120] Rauchman S. H. Zubair A. Jacob B. Rauchman D. Pinkhasov A. Placantonakis D. G. (2023). Traumatic brain injury: mechanisms, manifestations, and visual sequelae. Front. Neurosci. 17, 1090672. 10.3389/fnins.2023.1090672 36908792 PMC9995859

[B121] Rebenda D. Vrbka M. Čípek P. Toropitsyn E. Nečas D. Pravda M. (2020). On the dependence of rheology of hyaluronic acid solutions and frictional behavior of articular cartilage. Mater. (Basel) 13 (11), 2659. 10.3390/ma13112659 PMC7321645 32545213

[B122] Richa Choudhury A. R. (2020). pH mediated rheological modulation of chitosan hydrogels. Int. J. Biol. Macromol. 156, 591–597. 10.1016/j.ijbiomac.2020.04.049 32289416

[B123] Ripley D. L. Gerber D. Pretz C. Weintraub A. H. Wierman M. E. (2020). Testosterone replacement in hypogonadal men during inpatient rehabilitation following traumatic brain injury: results from a double-blind, placebo-controlled clinical pilot study. NeuroRehabilitation 46 (3), 355–368. 10.3233/nre-192992 32250330

[B124] Rizzo F. Kehr N. S. (2021). Recent advances in injectable hydrogels for controlled and local drug delivery. Adv. Healthc. Mater 10 (1), e2001341. 10.1002/adhm.202001341 33073515

[B125] Rodney T. Osier N. Gill J. (2018). Pro- and anti-inflammatory biomarkers and traumatic brain injury outcomes: a review. Cytokine 110, 248–256. 10.1016/j.cyto.2018.01.012 29396048

[B126] Rowland M. J. Parkins C. C. McAbee J. H. Kolb A. K. Hein R. Loh X. J. (2018). An adherent tissue-inspired hydrogel delivery vehicle utilised in primary human glioma models. Biomaterials 179, 199–208. 10.1016/j.biomaterials.2018.05.054 30037456

[B127] Saraiva C. Praça C. Ferreira R. Santos T. Ferreira L. Bernardino L. (2016). Nanoparticle-mediated brain drug delivery: overcoming blood-brain barrier to treat neurodegenerative diseases. J. Control Release 235, 34–47. 10.1016/j.jconrel.2016.05.044 27208862

[B128] Sarrigiannidis S. O. Rey J. Dobre O. González-García C. Dalby M. Salmeron-Sanchez M. (2021). A tough act to follow: collagen hydrogel modifications to improve mechanical and growth factor loading capabilities. Mater. Today Bio 10, 100098. 10.1016/j.mtbio.2021.100098 PMC7973388 33763641

[B129] Schimmel S. J. Acosta S. Lozano D. (2017). Neuroinflammation in traumatic brain injury: a chronic response to an acute injury. Brain Circ. 3 (3), 135–142. 10.4103/bc.bc_18_17 30276315 PMC6057689

[B130] Shapiro J. M. Oyen M. L. (2013). Hydrogel composite materials for tissue engineering scaffolds. JOM 65 (4), 505–516. 10.1007/s11837-013-0575-6

[B131] Shi K. Zhang J. Dong J. f. Shi F. D. (2019). Dissemination of brain inflammation in traumatic brain injury. Cell Mol. Immunol. 16 (6), 523–530. 10.1038/s41423-019-0213-5 30846842 PMC6804599

[B132] Shichinohe H. Kuroda S. Yano S. Hida K. Iwasaki Y. (2007). Role of SDF-1/CXCR4 system in survival and migration of bone marrow stromal cells after transplantation into mice cerebral infarct. Brain Res. 1183, 138–147. 10.1016/j.brainres.2007.08.091 17976542

[B133] Siegel R. M. Ka-Ming Chan F. Chun H. J. Lenardo M. J. (2000). The multifaceted role of Fas signaling in immune cell homeostasis and autoimmunity. Nat. Immunol. 1 (6), 469–474. 10.1038/82712 11101867

[B134] Simon D. W. McGeachy M. J. Bayır H. Clark R. S. B. Loane D. J. Kochanek P. M. (2017). The far-reaching scope of neuroinflammation after traumatic brain injury. Nat. Rev. Neurol. 13 (3), 171–191. 10.1038/nrneurol.2017.13 28186177 PMC5675525

[B135] Singh I. N. Sullivan P. G. Deng Y. Mbye L. H. Hall E. D. (2006). Time course of post-traumatic mitochondrial oxidative damage and dysfunction in a mouse model of focal traumatic brain injury: implications for neuroprotective therapy. J. Cereb. Blood Flow. Metab. 26 (11), 1407–1418. 10.1038/sj.jcbfm.9600297 16538231

[B136] Spada A. Emami J. Tuszynski J. A. Lavasanifar A. (2021). The uniqueness of albumin as a carrier in nanodrug delivery. Mol. Pharm. 18 (5), 1862–1894. 10.1021/acs.molpharmaceut.1c00046 33787270

[B137] Strong M. J. West G. A. Woo H. Couture D. E. Wilson J. A. (2017). A pivotal randomized clinical trial evaluating the safety and effectiveness of a novel hydrogel dural sealant as an adjunct to dural repair. Oper. Neurosurg. Hagerst. 13 (2), 204–212. 10.1093/ons/opw004 28927211

[B138] Subramanian S. M. (2020). Mechanical properties of materials: definition, testing and application. Int. J. Mod. Stud. Mech. Eng. 6, 28–38. 10.20431/2454-9711.0602003

[B139] Suhail M. Rosenholm J. M. Minhas M. U. Badshah S. F. Naeem A. Khan K. U. (2019). Nanogels as drug-delivery systems: a comprehensive overview. Ther. Deliv. 10 (11), 697–717. 10.4155/tde-2019-0010 31789106

[B140] Takahashi T. Marushima A. Nagasaki Y. Hirayama A. Muroi A. Puentes S. (2020). Novel neuroprotection using antioxidant nanoparticles in a mouse model of head trauma. J. Trauma Acute Care Surg. 88 (5), 677–685. 10.1097/ta.0000000000002617 32039974

[B141] Tang S. Gao P. Chen H. Zhou X. Ou Y. He Y. (2020). The role of iron, its metabolism and ferroptosis in traumatic brain injury. Front. Cell Neurosci. 14, 590789. 10.3389/fncel.2020.590789 33100976 PMC7545318

[B142] Tani J. Wen Y. T. Hu C. J. Sung J. Y. (2022). Current and potential pharmacologic therapies for traumatic brain injury. Pharm. (Basel) 15 (7), 838. 10.3390/ph15070838 PMC9323622 35890136

[B143] Tanikawa S. Ebisu Y. Sedlačík T. Semba S. Nonoyama T. Kurokawa T. (2023). Engineering of an electrically charged hydrogel implanted into a traumatic brain injury model for stepwise neuronal tissue reconstruction. Sci. Rep. 13 (1), 2233. 10.1038/s41598-023-28870-z 36788295 PMC9929269

[B144] Tarudji A. W. Gee C. C. Romereim S. M. Convertine A. J. Kievit F. M. (2021). Antioxidant thioether core-crosslinked nanoparticles prevent the bilateral spread of secondary injury to protect spatial learning and memory in a controlled cortical impact mouse model of traumatic brain injury. Biomaterials 272, 120766. 10.1016/j.biomaterials.2021.120766 33819812 PMC8068673

[B145] Tarudji A. W. Miller H. A. Curtis E. T. Porter C. L. Madsen G. L. Kievit F. M. (2023). Sex-based differences of antioxidant enzyme nanoparticle effects following traumatic brain injury. J. Control Release 355, 149–159. 10.1016/j.jconrel.2023.01.065 36720285 PMC10006352

[B146] Thapa K. Khan H. Singh T. G. Kaur A. (2021). Traumatic brain injury: mechanistic insight on pathophysiology and potential therapeutic targets. J. Mol. Neurosci. 71 (9), 1725–1742. 10.1007/s12031-021-01841-7 33956297

[B147] Timofeev I. Nortje J. Al-Rawi P. G. Hutchinson P. J. Gupta A. K. (2013). Extracellular brain pH with or without hypoxia is a marker of profound metabolic derangement and increased mortality after traumatic brain injury. J. Cereb. Blood Flow. Metab. 33 (3), 422–427. 10.1038/jcbfm.2012.186 23232949 PMC3587815

[B148] Tseng T. C. Tao L. Hsieh F. Wei Y. Chiu I. Hsu S. (2015). An injectable, self-healing hydrogel to repair the central nervous system. Adv. Mater 27 (23), 3518–3524. 10.1002/adma.201500762 25953204

[B149] Ulbrich K. Hekmatara T. Herbert E. Kreuter J. (2009). Transferrin- and transferrin-receptor-antibody-modified nanoparticles enable drug delivery across the blood-brain barrier (BBB). Eur. J. Pharm. Biopharm. 71 (2), 251–256. 10.1016/j.ejpb.2008.08.021 18805484

[B150] Visser K. Koggel M. Blaauw J. van der Horn H. J. Jacobs B. van der Naalt J. (2022). Blood-based biomarkers of inflammation in mild traumatic brain injury: a systematic review. Neurosci. Biobehav Rev. 132, 154–168. 10.1016/j.neubiorev.2021.11.036 34826510

[B151] Waggoner L. E. Kang J. Zuidema J. M. Vijayakumar S. Hurtado A. A. Sailor M. J. (2022). Porous silicon nanoparticles targeted to the extracellular matrix for therapeutic protein delivery in traumatic brain injury. Bioconjug Chem. 33 (9), 1685–1697. 10.1021/acs.bioconjchem.2c00305 36017941 PMC9492643

[B152] Wagner A. M. Gran M. P. Peppas N. A. (2018). Designing the new generation of intelligent biocompatible carriers for protein and peptide delivery. Acta Pharm. Sin. B 8 (2), 147–164. 10.1016/j.apsb.2018.01.013 29719776 PMC5925450

[B153] Wang J. Yang X. Klemeš J. J. Tian K. Ma T. Sunden B. (2023). A review on nanofluid stability: preparation and application. Renew. Sustain. Energy Rev. 188, 113854. 10.1016/j.rser.2023.113854

[B154] Wang L. Zhang D. Ren Y. Guo S. Li J. Ma S. (2022a). Injectable hyaluronic acid hydrogel loaded with BMSC and NGF for traumatic brain injury treatment. Mater Today Bio 13, 100201. 10.1016/j.mtbio.2021.100201 PMC8733324 35024600

[B155] Wang Y. Penna V. Williams R. J. Parish C. L. Nisbet D. R. (2022b). A hydrogel as a bespoke delivery platform for stromal cell-derived factor-1. Gels 8 (4), 224. 10.3390/gels8040224 35448125 PMC9025061

[B156] Wassenaar J. W. Gaetani R. Garcia J. J. Braden R. L. Luo C. G. Huang D. (2016). Evidence for mechanisms underlying the functional benefits of a myocardial matrix hydrogel for post-MI treatment. J. Am. Coll. Cardiol. 67 (9), 1074–1086. 10.1016/j.jacc.2015.12.035 26940929 PMC4779189

[B157] Watts M. E. Pocock R. Claudianos C. (2018). Brain energy and oxygen metabolism: emerging role in normal function and disease. Front. Mol. Neurosci. 11, 216. 10.3389/fnmol.2018.00216 29988368 PMC6023993

[B158] Wei C. Li P. Liu L. Zhang H. Zhao T. Chen Y. (2023). Degradable poly(amino acid) vesicles modulate DNA-induced inflammation after traumatic brain injury. Biomacromolecules 24 (2), 909–920. 10.1021/acs.biomac.2c01334 36629517

[B159] Wolak D. J. Thorne R. G. (2013). Diffusion of macromolecules in the brain: implications for drug delivery. Mol. Pharm. 10 (5), 1492–1504. 10.1021/mp300495e 23298378 PMC3646902

[B160] Wu D. Chen Q. Chen X. Han F. Chen Z. Wang Y. (2023a). The blood-brain barrier: structure, regulation, and drug delivery. Signal Transduct. Target Ther. 8 (1), 217. 10.1038/s41392-023-01481-w 37231000 PMC10212980

[B161] Wu M. Han L. Yan B. Zeng H. (2023b). Self-healing hydrogels based on reversible noncovalent and dynamic covalent interactions: a short review. Supramol. Mater. 2, 100045. 10.1016/j.supmat.2023.100045

[B162] Xiao H. Amarsaikhan O. Zhao Y. Yu X. Hu X. Han S. (2023). Astrocyte-targeted siRNA delivery by adenosine-functionalized LNP in mouse TBI model. Mol. Ther. Nucleic Acids 34, 102065. 10.1016/j.omtn.2023.102065 38028196 PMC10661454

[B163] Xiao H. Bao X. Bai N. Zhu W. Saqirila S. Hu X. (2024). Synthesis of lipidated ligands and formulation of glia-specific LNPs for RNAi-mediated BBB protection. J. Med. Chem. 67 (15), 13217–13230. 10.1021/acs.jmedchem.4c01176 39031092

[B164] Xie B. Xie H. (2024). Application of stimuli-responsive hydrogel in brain disease treatment. Front. Bioeng. Biotechnol. 12, 1450267. 10.3389/fbioe.2024.1450267 39091971 PMC11291207

[B165] Xiong B. Wang Y. Chen Y. Xing S. Liao Q. Chen Y. (2021). Strategies for structural modification of small molecules to improve blood-brain barrier penetration: a recent perspective. J. Med. Chem. 64 (18), 13152–13173. 10.1021/acs.jmedchem.1c00910 34505508

[B166] Xue Y. Ding J. Liu Y. Pan Y. Zhao P. Ren Z. (2020). Preparation and evaluation of recombinant human erythropoietin loaded tween 80-albumin nanoparticle for traumatic brain injury treatment. Int. J. Nanomedicine 15, 8495–8506. 10.2147/ijn.s264025 33154639 PMC7608583

[B167] Yang Y. Du T. Zhang J. Kang T. Luo L. Tao J. (2017). A 3D-engineered conformal implant releases DNA nanocomplexs for eradicating the postsurgery residual glioblastoma. Adv. Sci. (Weinh) 4 (8), 1600491. 10.1002/advs.201600491 28852611 PMC5566247

[B168] Youn D. H. Tran N. M. Kim B. J. Kim Y. Jeon J. P. Yoo H. (2021). Shape effect of cerium oxide nanoparticles on mild traumatic brain injury. Sci. Rep. 11 (1), 15571. 10.1038/s41598-021-95057-9 34330990 PMC8324865

[B169] Yu S. Xu X. Feng J. Liu M. Hu K. (2019). Chitosan and chitosan coating nanoparticles for the treatment of brain disease. Int. J. Pharm. 560, 282–293. 10.1016/j.ijpharm.2019.02.012 30772458

[B170] Zerbinati N. Sommatis S. Maccario C. Capillo M. C. Grimaldi G. Alonci G. (2021). Toward physicochemical and rheological characterization of different injectable hyaluronic acid dermal fillers cross-linked with polyethylene glycol diglycidyl ether. Polym. (Basel) 13 (6), 948. 10.3390/polym13060948 PMC8003446 33808730

[B171] Zhang D. Ren Y. He Y. Chang R. Guo S. Ma S. (2022). *In situ* forming and biocompatible hyaluronic acid hydrogel with reactive oxygen species-scavenging activity to improve traumatic brain injury repair by suppressing oxidative stress and neuroinflammation. Mater Today Bio 15, 100278. 10.1016/j.mtbio.2022.100278 PMC9119840 35601897

[B172] Zhang H. Zhu Y. Qu L. Wu H. Kong H. Yang Z. (2018b). Gold nanorods conjugated porous silicon nanoparticles encapsulated in calcium alginate nano hydrogels using microemulsion templates. Nano Lett. 18 (2), 1448–1453. 10.1021/acs.nanolett.7b05210 29382198

[B173] Zhang J. Allardyce B. J. Rajkhowa R. Zhao Y. Dilley R. J. Redmond S. L. (2018a). 3D printing of silk particle-reinforced chitosan hydrogel structures and their properties. ACS Biomater. Sci. Eng. 4 (8), 3036–3046. 10.1021/acsbiomaterials.8b00804 33435023

[B174] Zhang L. Fan J. Li G. Yin Z. Fu B. M. (2020). Transcellular model for neutral and charged nanoparticles across an *in vitro* blood-brain barrier. Cardiovasc Eng. Technol. 11 (6), 607–620. 10.1007/s13239-020-00496-6 33113565 PMC7592456

[B175] Zhang M. Shan H. Chang P. Wang T. Dong W. Chen X. (2014). Hydrogen sulfide offers neuroprotection on traumatic brain injury in parallel with reduced apoptosis and autophagy in mice. PLoS One 9 (1), e87241. 10.1371/journal.pone.0087241 24466346 PMC3900713

[B176] Zhang X. Huang X. Hang D. Jin J. Li S. Zhu Y. (2024). Targeting pyroptosis with nanoparticles to alleviate neuroinflammatory for preventing secondary damage following traumatic brain injury. Sci. Adv. 10 (2), eadj4260. 10.1126/sciadv.adj4260 38198543 PMC10780956

[B177] Zhang Y. Ma J. Zhang W. (2021). Berberine for bone regeneration: therapeutic potential and molecular mechanisms. J. Ethnopharmacol. 277, 114249. 10.1016/j.jep.2021.114249 34058315

[B178] Zheng Y. Wu G. Chen L. Zhang Y. Luo Y. (2021). Neuro-regenerative imidazole-functionalized GelMA hydrogel loaded with hAMSC and SDF-1α promote stem cell differentiation and repair focal brain injury. Bioact. Mater 6 (3), 627–637. 10.1016/j.bioactmat.2020.08.026 33005827 PMC7508914

[B179] Zhou G. Cao Y. Yan Y. Xu H. Zhang X. Yan T. (2024). Injectable hydrogels based on hyaluronic acid and gelatin combined with salvianolic acid B and vascular endothelial growth factor for treatment of traumatic brain injury in mice. Molecules 29 (8), 1705. 10.3390/molecules29081705 38675525 PMC11052029

[B180] Zhou T. Kalanuria A. (2018). Cerebral microdialysis in neurocritical care. Curr. Neurol. Neurosci. Rep. 18 (12), 101. 10.1007/s11910-018-0915-6 30353361

[B181] Zhou Y. Peng Z. Seven E. S. Leblanc R. M. (2018). Crossing the blood-brain barrier with nanoparticles. J. Control Release 270, 290–303. 10.1016/j.jconrel.2017.12.015 29269142

[B182] Zhou Z. W. Li F. Zheng Z. Li Y. Chen T. Gao W. (2017). Erythropoietin regulates immune/inflammatory reaction and improves neurological function outcomes in traumatic brain injury. Brain Behav. 7 (11), e00827. 10.1002/brb3.827 29201540 PMC5698857

[B183] Zhuang Z. Liu M. Luo J. Zhang X. Dai Z. Zhang B. (2022). Exosomes derived from bone marrow mesenchymal stem cells attenuate neurological damage in traumatic brain injury by alleviating glutamate-mediated excitotoxicity. Exp. Neurol. 357, 114182. 10.1016/j.expneurol.2022.114182 35901975

